# Tomato SlSAP3*,* a member of the stress‐associated protein family, is a positive regulator of immunity against *Pseudomonas syringae *pv. *tomato* DC3000

**DOI:** 10.1111/mpp.12793

**Published:** 2019-03-25

**Authors:** Shixia Liu, Jiali Wang, Siyu Jiang, Hui Wang, Yizhou Gao, Huijuan Zhang, Dayong Li, Fengming Song

**Affiliations:** ^1^ National Key Laboratory for Rice Biology, Institute of Biotechnology Zhejiang University Hangzhou Zhejiang 310058 China; ^2^ College of Life Science Taizhou University Taizhou Zhejiang 318000 China

**Keywords:** immune response, NudC, *Pseudomonas syringae* pv. *tomato* DC3000, stress‐associated proteins, tomato

## Abstract

Tomato stress‐associated proteins (SAPs) belong to A20/AN1 zinc finger protein family, some of which have been shown to play important roles in plant stress responses. However, little is known about the functions and underlying molecular mechanisms of SAPs in plant immune responses. In the present study, we reported the function of tomato SlSAP3 in immunity to *Pseudomonas syringae* pv. *tomato* (*Pst*) DC3000. Silencing of *SlSAP3* attenuated while overexpression of *SlSAP3* in transgenic tomato increased immunity to *Pst* DC3000, accompanied with reduced and increased *Pst *DC3000‐induced expression of SA signalling and defence genes, respectively. Flg22‐induced reactive oxygen species (ROS) burst and expression of PAMP‐triggered immunity (PTI) marker genes *SlPTI5* and *SlLRR22* were strengthened in *SlSAP3*‐OE plants but were weakened in *SlSAP3*‐silenced plants. SlSAP3 interacted with two SlBOBs and the A20 domain in SlSAP3 is critical for the SlSAP3‐SlBOB1 interaction. Silencing of *SlBOB1 *and co‐silencing of all three *SlBOB* genes conferred increased resistance to* Pst *DC3000, accompanied with increased *Pst *DC3000‐induced expression of SA signalling and defence genes. These data demonstrate that SlSAP3 acts as a positive regulator of immunity against *Pst* DC3000 in tomato through the SA signalling and that SlSAP3 may exert its function in immunity by interacting with other proteins such as SlBOBs, which act as negative regulators of immunity against *Pst* DC3000 in tomato.

## Introduction

Plants defend themselves against pathogen attack by deploying a multi‐layered immune system, which involve inducible defence responses and constitutive physical barriers (Jones and Dangl, 2006). The first layer of immune response is activated through detection of pathogen‐associated molecular patterns (PAMPs) by pattern recognition receptors (PRRs), which stimulate PAMP‐triggered immunity (PTI) (Boller and Felix, 2009; Macho and Zipfel, 2014; Schwessinger and Ronald, 2012). The second layer of immune response is often initiated after specific recognition of pathogen effectors by intracellular resistance (R) proteins, which are commonly known as effector‐triggered immunity (ETI) (Jones and Dangl, 2006). The defence responses associated with PTI and ETI may share some common signalling components and often trigger several early defence responses to restrict pathogen growth and spread (Boller and Felix, 2009; Meng and Zhang, 2013).

Stress‐associated protein (SAP) family is characterized by the presence of A20/AN1 zinc‐finger domains and is highly conserved in all plant species (Giri *et al*., 2013). However, little is known about the function of A20/AN1 proteins in plant disease resistance. Accumulating evidence revealed an important role for SAPs in plant immunity. Overexpression of rice *OsSAP1* in tobacco resulted in enhanced resistance against virulent bacterial pathogen, accompanied with up‐regulated expression of defence genes (Kothari *et al*., 2016; Tyagi *et al*., 2014). By contrast, it was found that overexpression of Arabidopsis *AtSAP9*, which was induced by pathogen, PAMP molecules and phytohormones, led to increased susceptibility to non‐host pathogen *Pseudomonas syringae* pv. *phaseolicola*, indicating that AtSAP9 plays key roles in basal resistance (Kang *et al*., 2017). Recently, an orchid SAP protein Pha13 and its *Arabidopsis* homologue AtSAP5 were reported to serve as an important regulatory hub in plant antiviral immunity (Chang *et al*., 2018). It was shown that SAPs regulate various stress responses by modulating phytohormone signalling cascades, which are mediated by JA, SA, ET and ABA. Interestingly, Arabidopsis AtSAP5 and AtSAP9 and rice OsSAP7 and OsSAP11 prefer to negatively regulate phytohormone signalling pathways (Kang *et al*., 2013, 2017, 2013, 2017; Liu *et al*., 2011; Sharma *et al*., 2015), while Pha13 positively regulates the expression of two SA responsive genes *PhaRdR1* and *PhaGRX* (Chang *et al*., 2018).

The biochemical function of SAPs has been shown to be associated with the ubiquitin/26S proteasome (UPS)‐mediated proteolysis system through acting as E3 ligases or interaction with UPS components. It was found that AtSAP5, acting as E3 ubiquitin ligase, plays a role as a positive regulator of drought stress responses (Choi *et al*., 2012; Kang *et al*., 2011, 2013). Besides, some SAPs were found to interact with UPS components, such as ubiquitin receptors RAD23s, which are capable of targeting ubiquitylated substrates to UPS (Farmer *et al*., 2010; Fu *et al*., 2010; Saeki, 2017). For instance, Arabidopsis AtSAP5, *Prunus* PpSAP1 and orchid Pha13 were found to interact with polyubiquitinated proteins (Chang *et al*., 2018; Choi *et al*., 2012; Lloret *et al*., 2017). AtSAP9 was found to interact with RAD23b and RAD23d, which act as shuttling factors of ubiquitin conjugates (Farmer *et al*., 2010; Guzder *et al*., 1998; Kang *et al*., 2017).

Despite these recent studies, a clear scenery of the role of SAPs and the mechanism by which SAPs regulate plant stress responses remain elusive. There are 13 members in tomato SlSAP family (Solanke *et al*., 2009). In the present study, we performed functional analyses using virus‐induced gene silencing (VIGS) for the roles of tomato SlSAPs in disease resistance against *Pseudomonas syringae* pv. *tomato* (*Pst*) DC3000. We found that silencing of *SlSAP3* resulted in decreased resistance whereas overexpression of *SlSAP3* in transgenic tomato led to enhanced resistance to *Pst* DC3000, accompanied with decreased and increased *Pst *DC3000‐induced expression of SA signalling and defence genes, respectively. We also found that SlSAP3 interacted with SlBOBBER1 (SlBOB1) and SlBOBBER2 (SlBOB2), tomato orthologues of eukaryotic NudC domain proteins and that silencing of *SlBOB1* or co‐silencing of three *SlBOB* genes resulted in enhanced resistance to *Pst* DC3000. Our data demonstrated that SlSAP3 positively regulates immunity to *Pst* DC3000 through SA signalling, possibly via the UPS pathway through interacting with SlBOBs.

## Results

### Silencing of *SlSAP3 *resulted in reduced resistance to *Pst* DC3000

To examine the possible involvement of SlSAPs in disease resistance, we performed functional analyses by VIGS approach. For this purpose, a specific fragment for each *SlSAP* gene (Table S1, see Supporting Information) was chosen to generate VIGS constructs (Liu *et al*., 2002). The silencing efficiency for a target *SlSAP* gene was approximately 70% (Fig. S1A, see Supporting Information). Besides, silencing specificity of *SlSAP3* was also examined (Fig. S1B, see Supporting Information). The silencing efficiencies for each of the *SlSAP* genes and specificity for *SlSAP3* were satisfied for further experiments.

**Figure 1:**
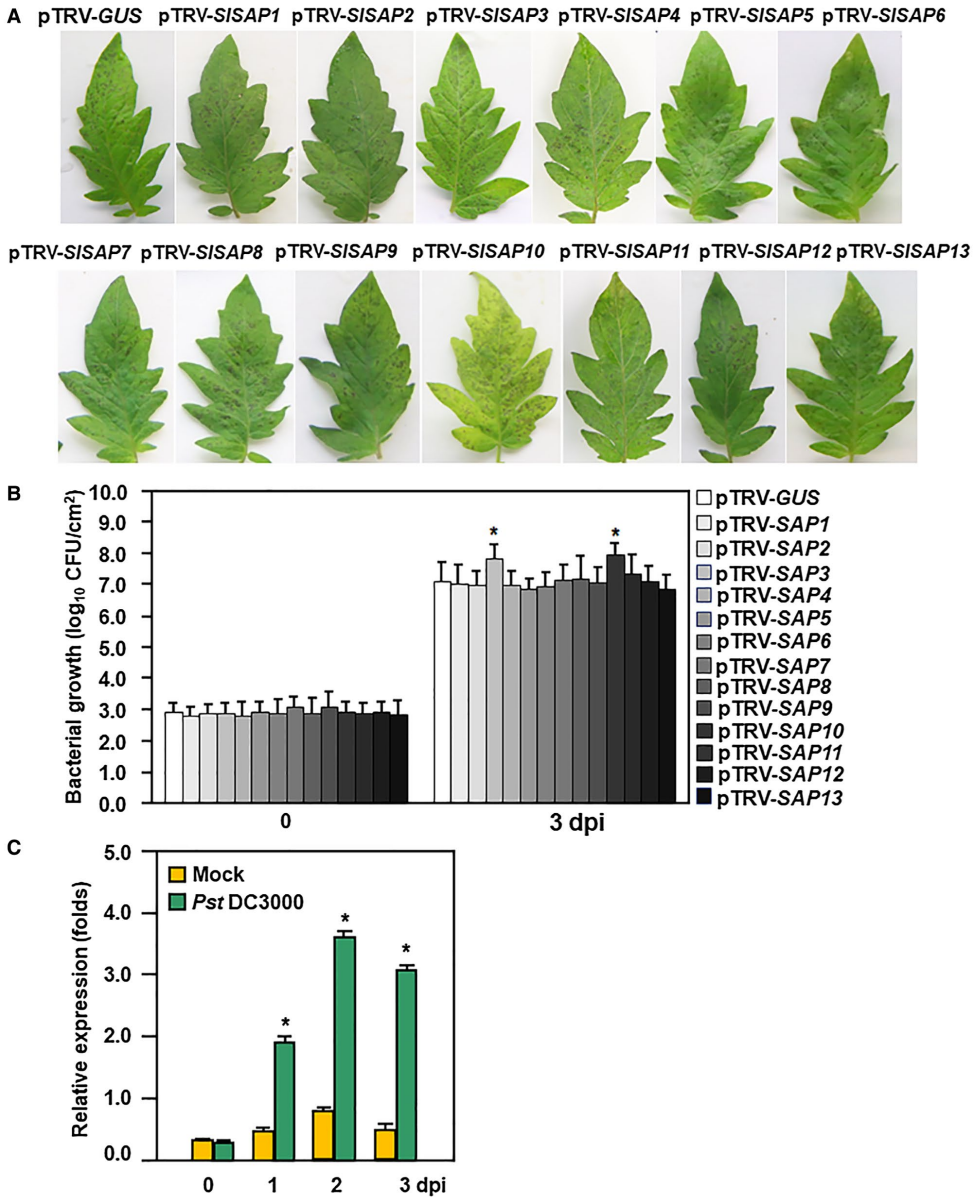
Attenuated resistance to *Pst* DC3000 in *SlSAP3‐* and *SlSAP10*‐silenced plants and responsiveness of *SlSAP3* to *Pst* DC3000. (A) Disease symptom and (B) bacterial populations in inoculated leaves at 3 dpi. Two‐week‐old seedlings were infiltrated with agrobacteria carrying pTRV‐*SlSAPs* or pTRV‐*GUS* and the agroinfiltrated plants were inoculated at 4 weeks after agroinfiltration by vacuum infiltration with *Pst* DC3000 suspension (OD_600 _= 0.0002). Photographs were taken and bacterial population was measured at 3 dpi. (C) Expression of *SlSAP3* in response to *Pst* DC3000. Four‐week‐old tomato plants were inoculated by spraying with *Pst* DC3000 suspension (OD_600_ = 0.2) or sterilized 10 mM MgCl_2_ solution as a mock‐inoculation control. Leaf samples were collected at indicated time points for analysis of gene expression. *SlActin* was used as an internal reference gene and relative expression was shown as folds of the transcript value of the *SlActin* gene. Data presented (B) and (C) are the means ± standard errors (SE) from three independent experiments. Statistical significance compared with pTRV‐*GUS* or mock‐inoculated plants was determined by Student's *t*‐tests: *P < 0.05. All experiments were repeated three times with similar results.

Next, we examined the changes in resistance of these pTRV‐*SlSAPs*‐infiltrated tomato plants to *Pst* DC3000. In our experiments, necrotic lesions on leaves of the pTRV‐*SlSAP3*‐infiltrated and pTRV‐*SlSAP10*‐infiltrated plants were larger and denser than those in the pTRV‐*GUS*‐infiltrated plants (Fig. 1A). At 3 days post‐inoculation (dpi), the bacterial population in the inoculated leaves of the pTRV‐*SlSAP3*‐infiltrated and pTRV‐*SlSAP10*‐infiltrated plants showed approximately eightfold and 10‐fold higher over those in the pTRV‐*GUS*‐infiltrated plants, respectively (Fig. 1B). These results indicate that silencing of either *SlSAP3* or *SlSAP10* resulted in reduced resistance to *Pst* DC3000. The pTRV‐*SlSAP10*‐infiltrated plants showed an earlier yellowing and senescent phenotype and thus, we focused on SlSAP3 to explore its function and mechanism in immune response against *Pst* DC3000.

The responsiveness of *SlSAP3* to *Pst* DC3000 was also examined. As shown in Fig. 1C, the expression level of *SlSAP3* in *Pst* DC3000‐infected plants started to increase at 1 dpi and gradually increased over a period of 3 days. These results indicate that *SlSAP3* responds to *Pst* DC3000.

### Overexpression of *SlSAP3* led to increased resistance against *Pst* DC3000

**Figure 2:**
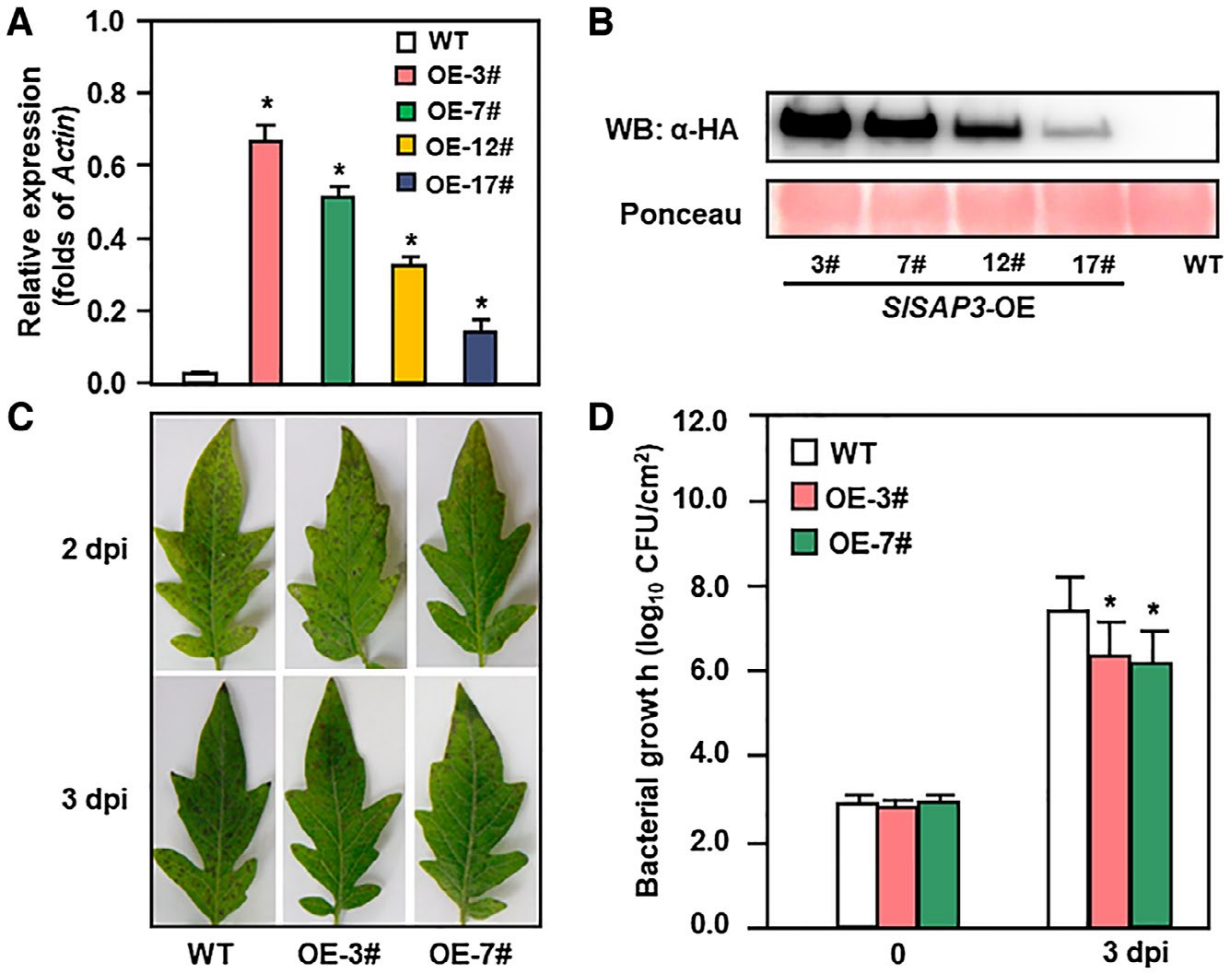
Enhanced *Pst* DC3000 resistance in *SlSAP3*‐OE plants. (A) Expression level of *SlSAP3* in wild type (WT) and four independent *SlSAP3*‐OE lines. Relative expression level was shown as folds of the transcript value of *Actin* gene. (B) Western blotting detection of SlSAP3‐HA fusion protein in *SlSAP3*‐OE plants. Total proteins were resolved by SDS‐PAGE and probed with anti‐HA antibody. (C) Representative *Pst* DC3000‐cuased disease symptom. (D) Bacterial growth in inoculated leaves of WT and *SlSAP3*‐OE plants. Four‐week‐old plants were inoculated by vacuum infiltration with *Pst* DC3000 suspension (OD_600_ = 0.002). Photographs were taken at 2 dpi and 3 dpi and bacterial population was measured at 0 dpi and 3 dpi. Data presented (A) and (D) are the means ± standard errors (SE) from three independent experiments. Statistical significance compared with WT was determined by Student's *t*‐tests: *P < 0.05. All experiments were repeated three times with similar results.

To further confirm the function of SlSAP3 in tomato immunity against *Pst* DC3000, we transformed 35S promoter driven overexpression SlSAP3 construct fused at the C‐terminal with a HA tag (*35S:SlSAP3‐HA*) into tomato cv. Ailsa Craig by *Agrobacterium*‐mediated transformation (Howe *et al*., 1996). A total of 13 independent transgenic lines were initially obtained and four transgenic homozygous lines were isolated. The transcript levels of *SlSAP3 *in overexpression lines were validated by quantitative Reverse Transcription‐Polymerase Chain Reaction (qRT‐PCR) (Fig. 2A), and the accumulation of SlSAP3‐HA fusion protein was detectable using antibody against HA tag (Fig. 2B). Two transgenic lines, *SlSAP3*‐OE‐3# and *SlSAP3*‐OE‐7#, were chosen for further studies as they had a relatively high level of expression of *SlSAP3*.

Disease phenotypic analyses showed that *Pst *DC3000‐caused lesions on leaves of *SlSAP3*‐OE plants were smaller and thinner than those in wild‐type (WT) plants (Fig. 2C). Accordingly, at 3 dpi, the bacterial population in the inoculated leaves of *SlSAP3*‐OE plants were significantly reduced as compared with those in WT plants (Fig. 2D). These results indicate that overexpression of *SlSAP3* intensified tomato resistance against *Pst* DC3000.

### Modification of *SlSAP3* expression affected *Pst *DC3000‐induced defence response

**Figure 3:**
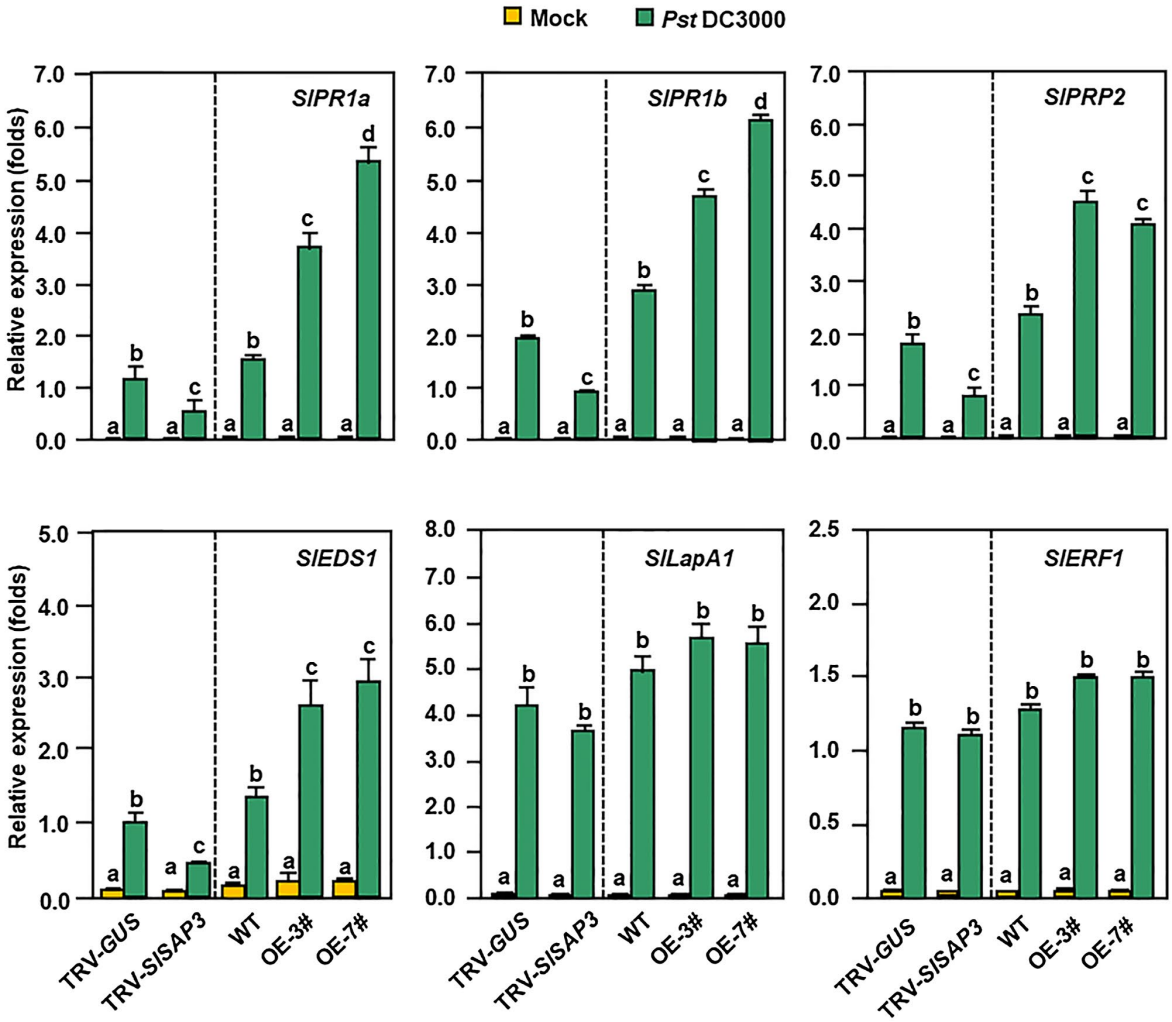
Expression patterns of signalling and defence genes in *SlSAP3*‐OE and *SlSAP3*‐silenced plants after *Pst *DC3000 infection. Four‐week‐old WT and *SlSAP3*‐OE plants or 5‐week‐old *SlSAP3*‐silenced and *GUS*‐silenced plants were inoculated by spraying with *Pst* DC3000 suspension (OD_600_ = 0.2) or with similar volume of buffer as mock‐inoculation controls. Leaf samples were collected at 24 h post‐inoculation (hpi) and expression of genes were analysed by quantitative Reverse Transcription‐Polymerase Chain Reaction (qRT‐PCR). *SlActin* was used as an internal reference gene and relative expression was shown as folds of the transcript value of the *SlActin* gene. Values represent means ± standard errors (SE) (*n* = 9) from three independent biological replicates and three technical replicates. Different letters indicate statistically significant differences. Multiple comparisons were calculated by one‐way analysis of variance (ANOVA) followed by Bonferroni post‐hoc test (*P* < 0.05). Data are representative of two independent experiments. MK, mock‐inoculated control; DC, *Pst* DC3000‐inoculated treatment.

To explore whether modification of *SlSAP3* expression affected the pathogen‐induced defence response, we analysed and compared the expression of defence genes after infection by *Pst* DC3000. As is shown in Fig. 3, the expression levels of some selected defence genes including *SlPR1a*, *SlPR1b*, *SlPR‐P2*, *SlEDS1*, *SlLapA1* and *SlERF1 *in *SlSAP3*‐OE and pTRV‐*SlSAP3*‐infiltrated plants were comparable to those in WT or pTRV‐*GUS*‐infiltrated plants without *Pst *DC3000 infection. At 24 h after infection by *Pst *DC3000, up‐regulated expression of *SlPR1a*, *SlPR1b*, *SlPR‐P2 *and *SlEDS1 *in *SlSAP3*‐OE plants were observed as compared with those in WT plants (Fig. 3). By contrast, down‐regulated expression of *SlPR1a*, *SlPR1b*, *SlPR‐P2 *and *SlEDS1* in pTRV‐*SlSAP3*‐infiltrated plants was detected as compared with those in pTRV‐*GUS*‐infiltrated plants, at 24 h after *Pst *DC3000 infection (Fig. 3). However, the *Pst* DC3000‐induced expression levels of *SlLapA1 *and *SlERF1* in *SlSAP3*‐OE plants and in pTRV‐*SlSAP3*‐infiltrated plants were comparable to those in WT and pTRV‐*GUS*‐infiltrated plants, respectively, at 24 h after *Pst *DC3000 infection (Fig. 3). Taken together, these results indicate that modification of *SlSAP3* expression affects the *Pst* DC3000‐induced expression of defence genes and thereby modulates immunity against this bacterial pathogen.

### Modification of *SlSAP3* expression affected flg22‐triggered PTI response

**Figure 4:**
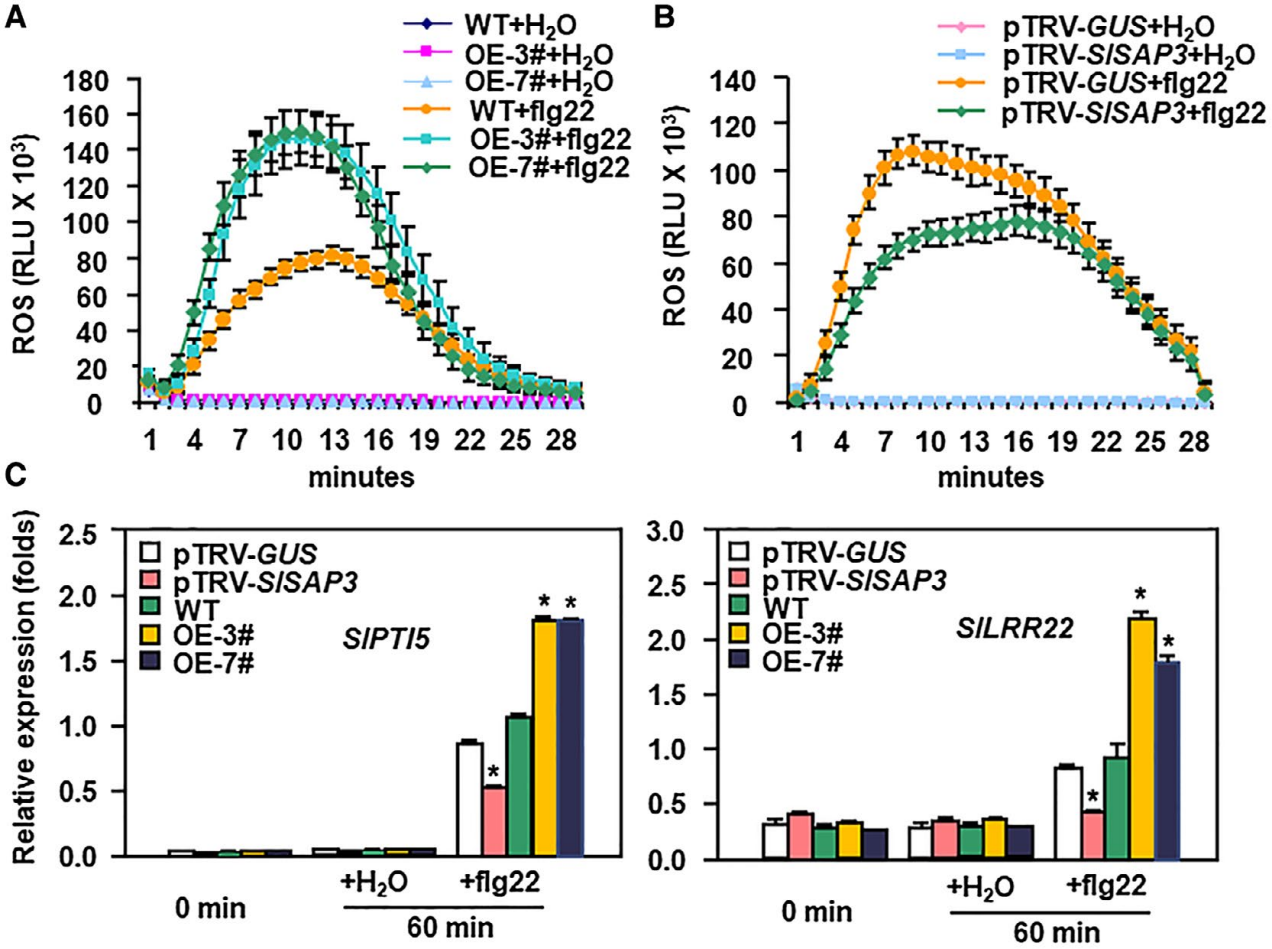
Altered flg22‐triggered immune response in *SlSAP3*‐OE and *SlSAP3*‐silenced plants. (A) flg22‐induced reactive oxygen species (ROS) burst in *SlSAP3*‐OE and wild‐type (WT) plants. (B) flg22‐induced ROS burst in *SlSAP3*‐silenced and *GUS*‐silenced plants. Leaf discs from 5‐week‐old plants were treated with water or 100 nM flg22 and ROS burst was monitored immediately over a period of 30 min after addition of flg22. Results are expressed as relative luminescence units (RLU). Experiments were repeated for three times with similar results. (C) Expression patterns of PTI marker genes. Leaf discs from 5‐week‐old plants were treated with 100 nM flg22 or water and harvested at 60 min after treatment for analyses of gene expression. *SlActin* was used as an internal reference gene and relative expression was shown as folds of the transcript value of the *SlActin* gene. Data presented in (C) are the means ± standard errors (SE) from three experiments with independent biological samples. Statistical significance compared with pTRV‐*GUS* and WT was determined by Student's *t*‐tests: *P < 0.05. All experiments were repeated three times with similar results.

To explore whether SlSAP3 is involved in tomato PTI response, we compared the flg22‐induced reactive oxygen species (ROS) burst and expression of PTI marker genes between *SlSAP3*‐OE and WT plants and between *SlSAP3*‐silenced and *GUS*‐silenced plants. In ROS burst assay, no significant ROS burst was detected in leaves of *SlSAP3*‐OE and WT plants and in leaves of pTRV‐*SlSAP3*‐ and pTRV‐*GUS*‐infiltrated plants without flg22 treatment (Fig. 4A,B). After addition of flg22, ROS burst in leaves of *SlSAP3*‐OE plants was relatively earlier and much enhanced as compared with that in WT plants (Fig. 4A). By contrast, a relatively lagged and significantly suppressed flg22‐induced ROS burst was observed in leaves of pTRV‐*SlSAP3*‐infiltrated plants as compared with that in pTRV‐*GUS*‐infiltrated plants (Fig. 4B).

Furthermore, the expression changes of *SlPTI5* and *SlLRR22*, two PTI marker genes in tomato (Kim *et al*., 2009; Nguyen *et al*., 2010), in *SlSAP3*‐OE plants and in pTRV‐*SlSAP3*‐infiltrated plants were also examined. At 60 min after treatment, the flg22‐induced expression of *SlPTI5* and *SlLRR22 *in leaves of *SlSAP3*‐OE and WT plants and in leaves of pTRV‐*SlSAP3*‐ and pTRV‐*GUS*‐infiltrated plants was detected (Fig. 4C). The flg22‐induced expression of *SlPTI5* and *SlLRR22* in leaves of *SlSAP3*‐OE plants was significantly strengthened as compared with that in WT plants (Fig. 4C). By contrast, the flg22‐induced expression of *SlPTI5* and *SlLRR22* was markedly suppressed in leaves of pTRV‐*SlSAP3*‐infiltrated plants as compared with those in pTRV‐*GUS*‐infiltrated plants (Fig. 4C). Collectively, these results indicate that overexpression of *SlSAP3 *strengthens while suppression of *SlSAP3 *partially attenuates the flg22‐induced PTI response.

### SlSAP3 did not possess ubiquitin E3 ligase activity *in vitro*

**Figure 5:**
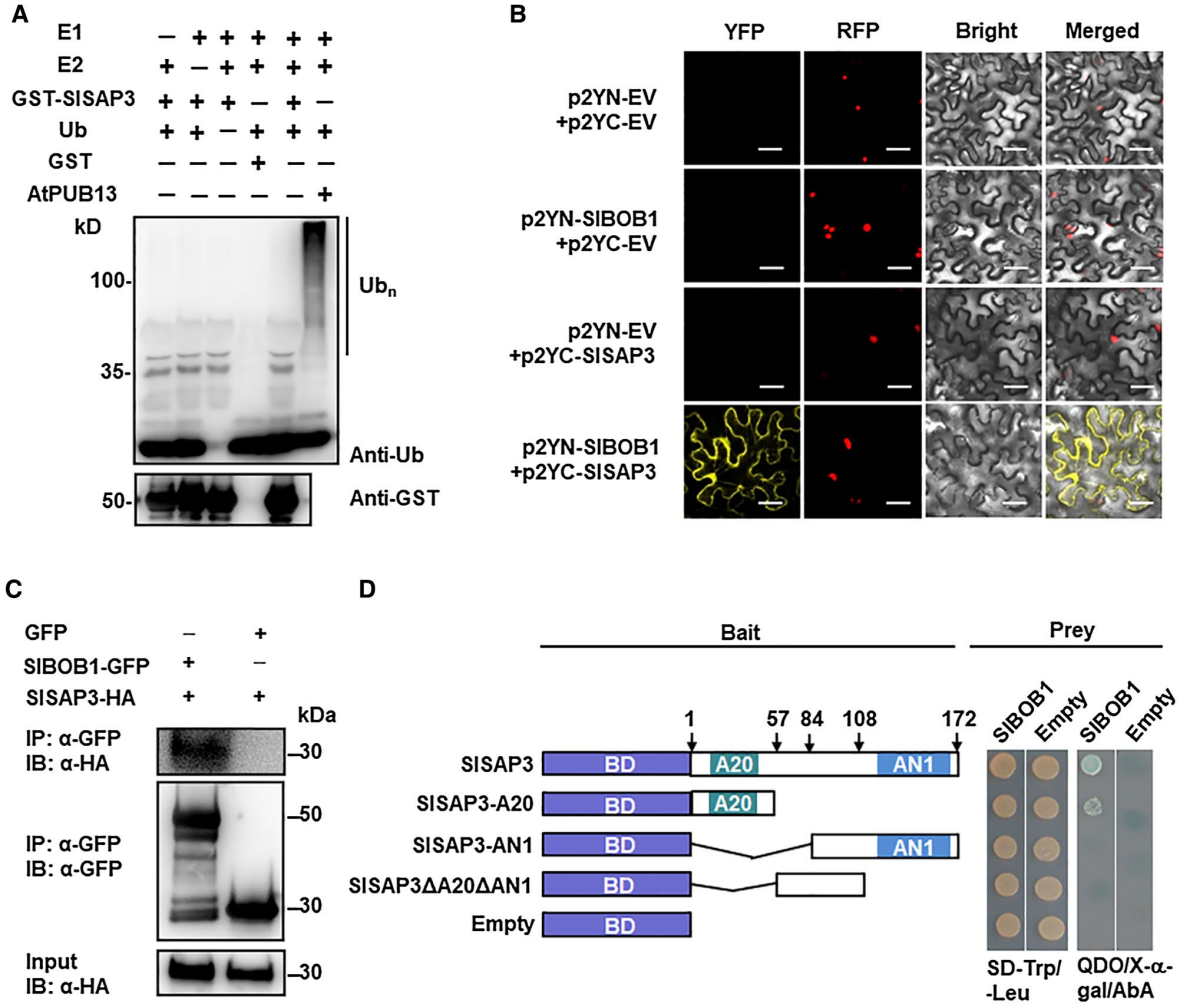
Biochemical activity of SlSAP3 and the interaction between SlSAP3 and SlBOB1. (A) SlSAP3 did not have ubiquitin E3 ligase activity *in vitro*. Ubiquitination reactions were performed at 30 ºC for 3 h, resolved by SDS‐PAGE and detected by immunoblotting using anti‐ubiquitin antibody. (B) Bimolecular fluorescence complementation (BiFC) analyses of *in planta* SlSAP3‐SlBOB1 interaction. Agrobacteria carrying different pairs of p2YC and p2YN plasmids were infiltrated into leaves of *Nicotiana benthamiana* and yellow florescent protein (YFP) signal was visualized under confocal microscopy at 48 h after infiltration. Bar = 50 µm. (C) Co‐immunoprecipitation (co‐IP) analyses of *in planta* SlSAP3‐SlBOB1 interaction. Agrobacteria harbouring SlSAP3‐HA and SlBOB1‐GFP were co‐infiltrated into *N. benthamiana* leaves and total proteins were extracted at 48 h after agroinfiltration. Immunoprecipitated proteins were separated on 12% SDS‐PAGE and were detected by immunoblotting with anti‐GFP‐specific antibody or anti‐HA‐specific antibody as indicated. (D) A20 domain in SlSAP3 is required for the SlSAP3‐SlBOB1 interaction. Different truncated mutants of SlSAP3 were generated (*left*) and examined for their interaction activity with SlBOB1 (*right*). Yeast cells co‐transformed with indicated pairs of pGBKT7 and pGADT7 vectors were incubated on SD/‐Trp/‐Leu and SD/‐Ade/‐His/‐Leu/‐Trp + X‐α‐gal + AbA (QDO/X‐α‐gal/AbA) plates and interaction activity was judged by the appearance of blue colour. Experiments in (A) and (B) were repeated for three times with similar results.

Several A20 domain‐containing proteins from animals and plants have been shown to possess E3 ubiquitin ligase activity (Kang *et al*., 2011, 2017, 2011, 2017; Wertz *et al*., 2004; Zhang *et al*., 2017). To determine whether SlSAP3 has E3 ubiquitin ligase activity, recombinant GST‐SlSAP3 protein was produced and tested for E3 ubiquitin ligase activity *in vitro*. In standard E3 ubiquitin ligase activity assays, no polyubiquitinated products were detected in reactions that lacked ubiquitin, yeast E1, human E2 or GST‐SlSAP3 while significant polyubiquitinated products were observed in the presence of ubiquitin, yeast E1, human E2 and a positive control Arabidopsis AtPUB13 (Liao *et al*., 2017) (Fig. 5A). However, in the presence of ubiquitin, yeast E1 and human E2, the GST‐SlSAP3 fusion protein failed to catalyze the formation of polyubiquitinated products (Fig. 5A). These results indicate that SlSAP3 may not possess E3 ubiquitin ligase activity *in vitro*.

### SlSAP3 interacted with SlBOB1

To further explore the molecular mechanism of SlSAP3 in tomato immunity against *Pst* DC3000, we tried to identify possible SlSAP3‐interactors. A cDNA library from *Pst *DC3000 infected tomato leaves was used as the prey, and the full‐length SlSAP3 was used as the bait. After screening 2 × 10^6^ yeast cells transformed with a cDNA library prepared from *Pst* DC3000‐infected tomato leaves, 35 positive clones were initially obtained. Of these, 21 clones contained in‐frame coding sequences coding for six proteins (Table S2, see Supporting Information). Amongst them, the putative SlSAP3‐interactor SlBOBBER1 (SlBOB1) (Solyc03g083390), which is a homologue of Arabidopsis non‐canonical small heat shock protein required for both development and abiotic stress response (Kahloul *et al*., 2013; Perez *et al*., 2009; Silverblatt‐Buser *et al*., 2018), was of our interest for further study. Bimolecular fluorescence complementation (BiFC) and co‐immunoprecipitation (co‐IP) assays were conducted to further verify the SlSAP3‐SlBOB1 interaction *in planta*. In BiFC assays, yellow florescent protein (YFP) signal was not detected in leaves co‐infiltrated with agrobacteria harbouring p2YN‐EV (empty vector) and p2YC‐EV (empty vector), p2YN‐SlBOB1 and p2YC‐EV and p2YN‐EV and p2YC‐SlSAP3, whereas significant YFP fluorescence was clearly observed in leaves co‐infiltrated with agrobacteria harbouring p2YN‐SlBOB1 and p2YC‐SlSAP3 (Fig. 5B). The constructs used for confocal observation were all successfully expressed, as revealed by protein gel blot analysis (Fig. S2, see Supporting Information). Notably, the fluorescence generated from the SlSAP3‐SlBOB1 interaction was observed in both nuclear and cytoplasmic compartments (Fig. 5B). Similarly, when transiently expressed in leaves of *N. benthamiana* plants, the GFP‐SlSAP3 and GFP‐SlBOB1 were localized in both nuclear and cytoplasmic compartments of epidermal cells (Fig. S3, see Supporting Information). Co‐IP assays in *N. benthamiana* after transient coexpression further confirmed that GFP‐SlBOB1 immunoprecipitated with SlSAP3‐HA but not with the empty vector expressing green florescent protein (GFP) alone (Fig. 5C). Taken together, these results demonstrated that SlSAP3 interacts with SlBOB1 *in planta*.

To determine the domains in SlSAP3 that are crucial for the SlSAP3‐SlBOB1 interaction, we created a series of truncated mutants of SlSAP3 (Fig. 5D). In Y2H assays, SlSAP3‐A20 did show interaction activity with SlBOB1, although the interaction activity of SlSAP3‐A20 seemed relatively weaker than the full‐length SlSAP3 (Fig. 5D). By contrast, SlSAP3‐AN1and SlSAP3ΔA20ΔAN1 completely abolished the interaction activity with SlBOB1 (Fig. 5D). These results indicate that the A20 domain in SlSAP3 is crucial for the SlSAP3‐SlBOB1 interaction.

### Silencing of *SlBOB1* resulted in increased resistance to *Pst* DC3000

**Figure 6:**
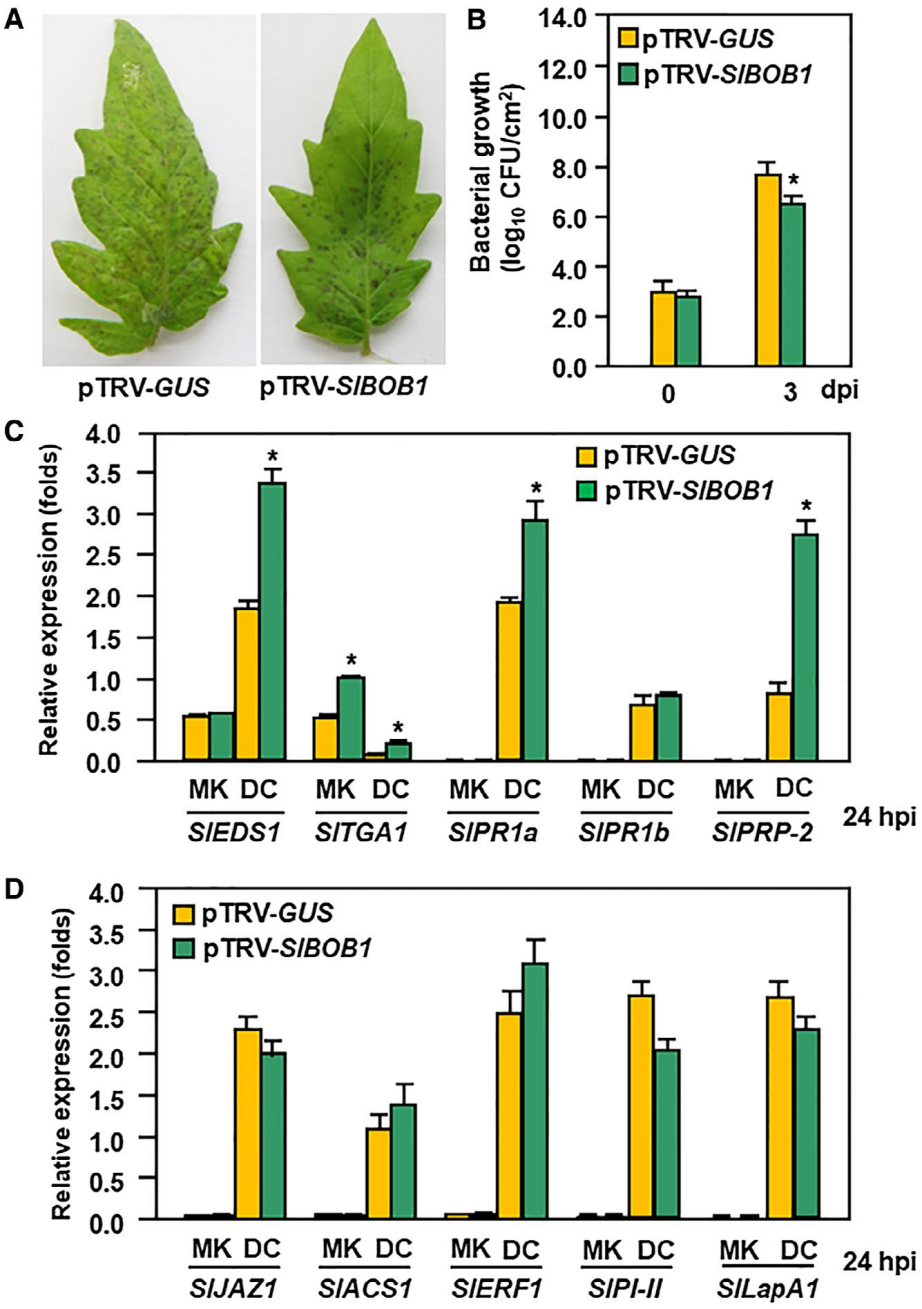
Enhanced *Pst* DC3000 resistance in *SlBOB1*‐silenced plants. (A) Representative disease symptom and (B) bacterial growth in inoculated leaves. Two‐week‐old seedlings were infiltrated with agrobacteria carrying pTRV‐*SlBOB1* or pTRV‐*GUS* and were inoculated by vacuum infiltration with *Pst* DC3000 suspension (OD_600 _= 0.002) at 4 weeks after agroinfiltration. Photographs were taken and bacterial population was measured at 3 dpi. (C) Expression of SA signalling and defence genes. (D) Expression of JA/ET signalling and defence genes. Two‐week‐old seedlings were infiltrated with agrobacteria carrying pTRV‐*SlBOB1* or pTRV‐*GUS* and were inoculated by spraying with *Pst* DC3000 suspension (OD_600_ = 0.2) or with similar volume of buffer as mock‐inoculation controls. Leaf samples were collected at 24 hpi and expression of genes were analysed by quatitative Reverse Transcription‐Polymerase Chain Reaction (qRT‐PCR). *SlActin* was used as an internal reference gene and relative expression was shown as folds of the transcript value of the SlActin gene. Data presented (B–D) are the means ± standard errors (SE) from three independent experiments. Statistical significance compared with pTRV‐*GUS* was determined by Student's *t*‐tests: *P < 0.05. All experiments were repeated three times with similar results. MK, mock‐inoculated control; DC, *Pst* DC3000‐inoculated treatment.

Because SlSAP3 interacted with SlBOB1, we then examined whether SlBOB1 played a role in tomato immunity against *Pst *DC3000. At 3 dpi, *Pst* DC3000‐caused necrotic lesions on leaves of pTRV‐*SlBOB1*‐infiltrated plants were less severe than that in pTRV‐*GUS*‐infiltrated plants (Fig. 6A) and pTRV‐*SlBOB1*‐infiltrated plants supported less bacterial population as compared with that in the inoculated leaves of pTRV‐*GUS*‐infiltrated plants (Fig. 6B). These results suggest that silencing of *SlBOB1* resulted in increased resistance to *Pst *DC3000.

To explore whether silencing of *SlBOB1* affected the *Pst* DC3000‐induced defence response, we analysed and compared the expression changes of signalling and defence genes in pTRV‐*SlBOB1*‐ and pTRV‐*GUS*‐infiltrated plants before and after *Pst* DC3000 infection. In mock‐inoculated pTRV‐*SlBOB1*‐ and pTRV‐*GUS*‐infiltrated plants, the expression levels of some selected regulatory genes of the SA and JA/ET signalling pathways and their corresponding defence genes were comparable except for an increase of expression of *SlTGA1* (Fig. 6C,D). The expression of the SA signalling regulatory genes *SlEDS1* and *SlTGA1* and the corresponding defence genes *SlPR1a *and *SlPR‐P2* in pTRV‐*SlBOB1*‐ and pTRV‐*GUS*‐infiltrated plants was significantly up‐regulated at 24 h after *Pst *DC3000 infection (Fig. 6C); however, the expression levels in pTRV‐*SlBOB1*‐infiltrated plants were higher than those in pTRV‐*GUS*‐infiltrated plants (Fig. 6C). By contrast, the expression levels of the SA signalling defence gene *SlPR1b*, the JA/ET signalling regulatory genes *SlJAZ1*, *SlACS1* and *SlERF1* and the corresponding defence genes *SlPI‐II* and *SlLapA1* were comparable in pTRV‐*SlBOB1*‐ and pTRV‐*GUS*‐infiltrated plants at 24 h after *Pst *DC3000 infection (Fig. 6C,D). These results indicate that silencing of *SlBOB1* enhanced the SA signalling and defence response upon infection of *Pst *DC3000. Taken together, these data suggest that SlBOB1, unlike the function of SlSAP3, negatively regulates immunity against *Pst *DC3000

### SlSAP3 interacted with SlBOB2 and co‐silencing of SlBOBs enhanced resistance to Pst DC3000

To test whether SlSAP3 interacted with other SlBOB proteins and whether they are also involved in immunity against *Pst* DC3000, we characterized the tomato SlBOB family and analysed the interaction of SlSAP3 with other SlBOB members. In addition to SlBOB1, two more SlBOB members were identified and named as SlBOB2 (Solyc02g062410) and SlBOB3 (Solyc06g051950), respectively (Table S4, see Supporting Information). Sequence alignment revealed that the three SlBOB proteins contain conserved characteristic NudC domain at their C‐terminals but the regions outside the NudC domain are divergent (Fig. S4A, see Supporting Information). Phylogenetic tree analyses indicated that SlBOB1 and SlBOB3 were clustered into one branch while SlBOB2 was closer to BOB proteins from Arabidopsis, *Brassica rapa*, rice and soybean (Fig. S4B, see Supporting Information).

**Figure 7:**
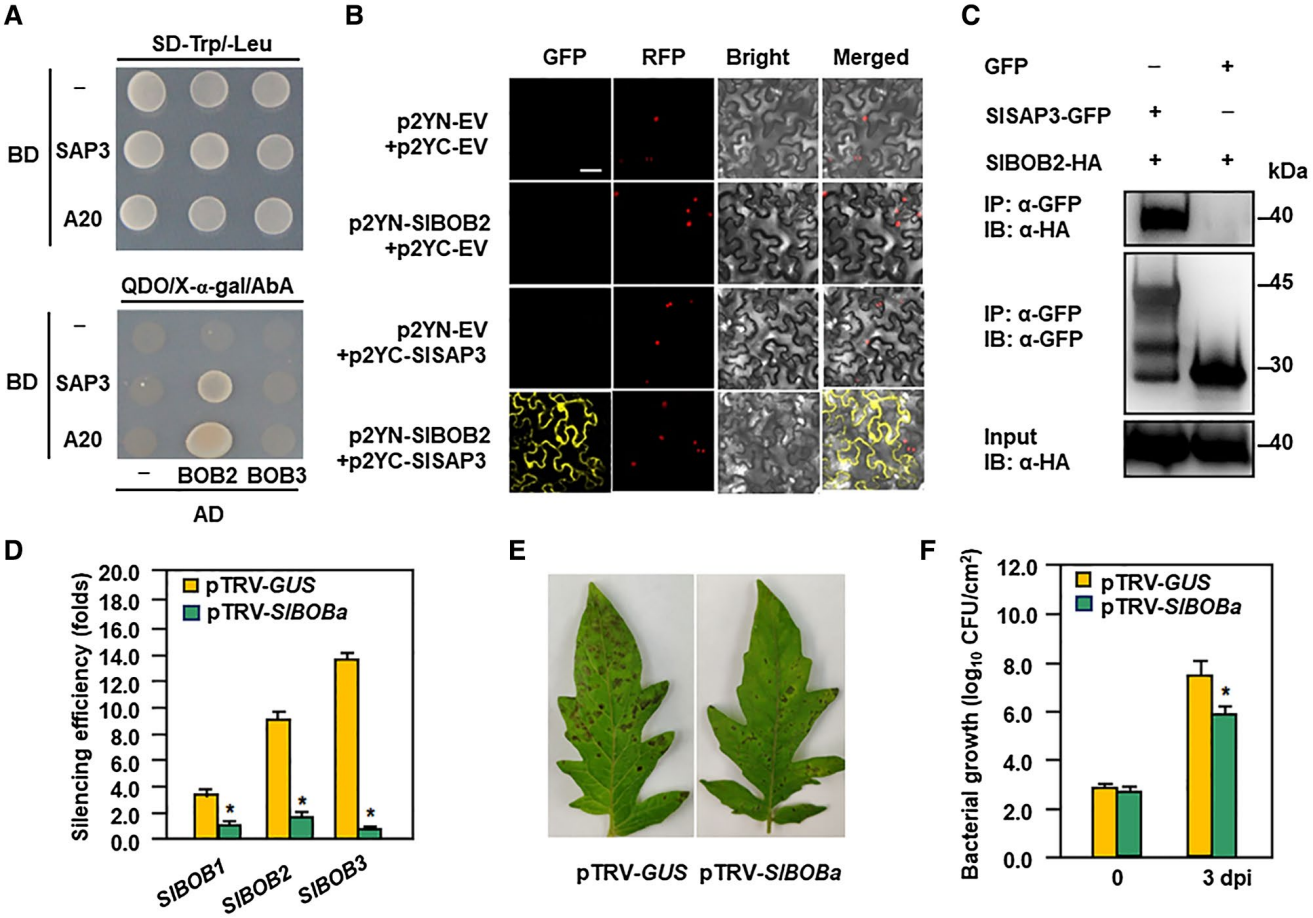
Interaction of SlSAP3 with SlBOB2 and enhanced *Pst* DC3000 resistance in *SlBOBs*‐co‐silenced plants. (A) SlSAP3 interacted with SlBOB2 but not with SlBOB3. Co‐transformed yeast cells were grown on SD/DDO (SD‐Leu‐Trp) medium (upper panel) and grown on SD/QDO/X/A (SD‐Leu‐Trp‐His‐Ade+X‐α‐Gal+Aureobasidin A) medium (lower panel). (B) Bimolecular fluorescence complementation (BiFC) analyses of *in planta* SlSAP3‐SlBOB2 interaction. Agrobacteria carrying different pairs of p2YC and p2YN plasmids were infiltrated into leaves of *Nicotiana benthamiana* and yellow fluorescent protein (YFP) signal was visualized under confocal microscopy at 48 h after infiltration. Bar = 50 µm. (C) Co‐immunoprecipitation (co‐IP) analysis of *in planta* SlSAP3‐SlBOB2 interaction. Agrobacteria harbouring SlSAP3‐GFP and SlBOB2‐HA were co‐infiltrated into *N. benthamiana* leaves and total proteins were extracted at 48 h after agroinfiltration. Immunoprecipitated proteins were separated on 12% SDS‐PAGE and were detected by immunoblotting with anti‐GFP‐specific antibody or anti‐HA‐specific antibody as indicated. (D) Silencing efficiency in *SlBOBs*‐co‐silenced plants. (E) Representative disease symptom and (F) bacterial growth in inoculated leaves. Two‐week‐old seedlings were infiltrated with agrobacteria carrying pTRV‐*SlBOBa* or pTRV‐*GUS* and were inoculated by vacuum infiltration with *Pst* DC3000 suspension (OD_600 _= 0.002) at weeks after agroinfiltration. Photographs were taken and bacterial population was measured at 3 dpi. Experiments in (A–C) and (E) were repeated for three times with similar results. Data presented (D) and (F) are the means ± standard errors (SE) from three independent experiments. Statistical significance compared with pTRV‐*GUS* was determined by Student's *t*‐tests: *P < 0.05. All experiments were repeated three times with similar results.

We examined the interaction of SlSAP3 with SlBOB2 and SlBOB3 by Y2H, BiFC and Co‐IP assays. In Y2H assays, SlBOB2 interacted with both the full SlSAP3 and the SlSAP3‐A20 while SlBOB3 did not (Fig. 7A). In BiFC assays, YFP fluorescence was not detected in leaves co‐infiltrated with agrobacteria harbouring p2YN‐EV and p2YC‐EV, p2YN‐SlBOB2 and p2YC‐EV and p2YN‐EV and p2YC‐SlSAP3, while significant YFP fluorescence was clearly observed in leaves co‐infiltrated with agrobacteria harbouring p2YN‐SlBOB2 and p2YC‐SlSAP3 (Fig. 7B). Notably again, YFP signal from the SlBOB2‐SlSAP3 interaction was observed in both nuclear and cytosolic compartments of epidermal cells (Fig. 7B). Co‐IP experiments in *N. benthamiana* after transient coexpression confirmed that GFP‐SlSAP3 immunoprecipitated with SlBOB2‐HA but not with the empty vector expressing GFP alone (Fig. 7C). Taken together, these results demonstrated that SlSAP3 interacts with SlBOB1 and SlBOB2 but not with SlBOB3 *in planta*.

Because the coding sequences of the *SlBOB* genes are highly conserved, a conserved fragment with high levels of sequence identity amongst *SlBOBs *(Fig. S4A and Table S1, see Supporting Information), designated as *SlBOBa*, was used to co‐silence all *SlBOB *genes. In pTRV‐*SlBOBa*‐infiltrated plants, the transcript levels of *SlBOB1*, *SlBOB2* and *SlBOB3 *were simultaneously and significantly reduced by 70%, 82% and 95%, respectively, as compared with those in pTRV‐*GUS*‐infiltrated plants, at 3 weeks after agroinfiltration (Fig. 7D). We then examined the changes of resistance in pTRV‐*SlBOBa*‐infiltrated plants. As shown in Fig. 7E, the *Pst* DC3000‐caused disease severity on leaves of pTRV‐*SlBOBa*‐infiltrated plants was less severe than that in pTRV‐*GUS*‐infiltrated plants. Accordingly, the pTRV‐*SlBOBa*‐infiltrated plants supported less bacterial growth as compared with that in pTRV‐*GUS*‐infiltrated plants, at 3 dpi after *Pst* DC3000 infection (Fig. 7F). These results indicate that co‐silencing of *SlBOBs *led to a further increase in tomato resistance against *Pst *DC3000.

## Discussion

Emerging evidence has indicated that SAPs are involved in plant immunity (Kang *et al*., 2017; Tyagi *et al*., 2014). In the present study, we found that SlSAP3 plays important roles in resistance to *Pst* DC3000, as silencing of *SlSAP3* attenuated while overexpression of *SlSAP3* strengthened resistance to *Pst* DC3000 (Figs 1 and 2) and modification of *SlSAP3* expression markedly affected the expression of *Pst *DC3000‐induced defence genes (Fig. 3). Furthermore, *SlSAP3* also has functions in tomato PTI response, as revealed by the changes in flg22‐induced ROS burst and PTI marker gene expression (Fig. 4). Interestingly, SlSAP3 interacted with SlBOB1 and SlBOB2, two of the three tomato SlBOB family members (Figs 5 and 7), and silencing of *SlBOB1* or co‐silencing of all *SlBOB* genes led to enhanced resistance to *Pst* DC3000 (Figs 6 and 7). These observations demonstrate that SlSAP3 acts as a positive regulator of immunity against *Pst* DC3000 in tomato, providing new insights into the biological function of plant SAPs.

It is generally accepted that immune response against (hemi)biotrophic pathogens such as *Pst* DC3000 is modulated through the SA signalling (Glazebrook, 2005; Grant and Jones, 2009; Mengiste, 2012; Verhage *et al*., 2010). The *Pst* DC3000‐induced expression of SA signalling regulatory gene *SlEDS1* and SA signalling‐responsive defence genes *SlPR1a*, *SlPR1b* and *SlPR‐P2* was strengthened in *SlSAP3‐*OE plants while partially suppressed in *SlSAP3*‐silenced plants (Fig. 3). By contrast, the expression of JA/ET signalling regulatory gene *SlERF1* and defence gene *SlLapA1* was not significantly affected by *Pst* DC3000 in both *SlSAP3‐*OE and *SlSAP3*‐silenced plants (Fig. 3). Such different expression patterns imply that the SA signalling pathway is required for the function of SlSAP3 in immunity against *Pst *DC3000. This is consistent with a recent observation that orchid SAP protein Pha13 positively regulates the expression of SA‐mediated immune responsive genes (Chang *et al*., 2018), but is different from a previous observation that AtSAP9 positively acts in JA signalling and negatively acts in SA pathway in response to a non‐host pathogen challenge (Kang *et al*., 2017).

ROS burst is an early response in PTI by serving as an anti‐microbial agent and/or as a secondary messenger that triggers downstream defence responses (Kadota *et al*., 2014; Mengiste, 2012). In our experiment, the flg22‐induced ROS burst was relatively earlier and much enhanced in leaves of *SlSAP3*‐OE plants while it was relatively lagged and significantly suppressed in leaves of *SlSAP3*‐silenced plant (Fig. 4A,B). Meanwhile, the flg22‐induced expression of PTI marker genes *SlPTI5* and *SlLRR22* was enhanced in *SlSAP3*‐OE plants but was weakened in *SlSAP3*‐silenced plants (Fig. 4C). These features demonstrate a function for SlSAP3, as a positive regulator, in tomato PTI. However, it was previously reported that overexpression of *AtSAP9* led to increased susceptibility to a non‐host bacterial pathogen, *P. syringae* pv. *phaseolicola*, indicating that AtSAP9 is a negative regulator of basal resistance (Kang *et al*., 2017). The reason for the opposite roles of SlSAP3 and AtSAP9 can be partially interpreted that they belong to different clades and may have functionally diverged during evolution based on phylogenetic analysis of SlSAP3 with other reported plant SAP proteins (Fig. S5, see Supporting Information). The phylogenetic analysis also revealed the multiple functions of SAPs as most of the clades contain members involved in plant biotic and abiotic stress responses (Fig. S5, see Supporting Information). Despite these contrary results, it seems clear that SAPs play roles in plant PTI/basal resistance.

It has been shown that some of the A20 domain‐containing proteins of animal origins possess ubiquitin E3 ligase activity (Mattera *et al*., 2006; Wertz *et al*., 2004). In plants, the Arabidopsis AtSAP5 and AtSAP9, rice OsiSAP7 and orchid Pha13, all of which contain both A20 and AN1 domains, were found to exhibit ubiquitin E3 ligase activity (Kang *et al*., 2011, 2017, 2011, 2017; Sharma *et al*., 2015). Furthermore, the A20 domains in AtSAP5 and Pha13 were responsible for both E3 ligase and ubiquitin binding ability (Chang *et al*., 2018; Kang *et al*., 2011). It was also reported that the AN1 domain in AtSAP5 had strong ubiquitin E3 ligase activity (Choi *et al*., 2012). Although SlSAP3 contains typical A20 and AN1 domains, we failed to detect the ubiquitin E3 ligase activity for SlSAP3 in our repeated experiments (Fig. 5A), in which the positive control AtPUB13 showed clear ubiquitin E3 ligase activity (Liao *et al*., 2017). It is therefore likely that ubiquitin E3 ligase activity may not be a common feature for the A20/AN1 domain‐containing SAPs. On the other hand, the Arabidopsis AtSAP5 and AtSAP9, *Prunus* PpSAP1 and orchid Pha13 were found to interact with polyubiquitinated proteins or with UPS shuttling factors such as RADs (Chang *et al*., 2018; Choi *et al*., 2012; Farmer *et al*., 2010; Kang *et al*., 2017; Lloret *et al*., 2017). The A20 domains in AtSAP5 and Pha13 are responsible for ubiquitin binding activity (Chang *et al*., 2018; Choi *et al*., 2012). In the present study, clones containing genes coding for ubiquitin or ubiquitin‐ribosomal fusion protein appeared with high frequency in our efforts towards identification of SlSAP3 interactors (Table S3, see Supporting Information), implying that SlSAP3 may interact with UPS components in nature. Thus, it is likely that SlSAP3 may exert its function in immunity by interacting with other proteins such as UPS components rather than by its ubiquitin E3 ligase activity.

It was previously reported that the Arabidopsis BOBs, acting as protein chaperones and interactors of multiple UPS subunits/components (Gunsalus *et al*., 2005; Perez *et al*., 2009; Zheng *et al*., 2011), are required for growth, development and abiotic stress responses (Jurkuta *et al*., 2009; Ishibashi *et al*., 2012; Perez *et al*., 2009; Silverblatt‐Buser *et al*., 2018). In the present study, SlBOB1 and one of its homologues SlBOB2 appeared as real SlSAP3 interacting partners *in planta*, as verified by BiFC and co‐IP approaches (Figs 5 and 7). It is clear that SlBOB1 plays a role in immunity against *Pst* DC3000, as the *SlBOB1*‐silenced plants displayed enhanced resistance and up‐regulated expression of defence genes upon pathogen infection (Fig. 6). However, *SlBOBs*‐co‐silenced plants showed less disease severity and supported less bacterial growth, as compared with that in *SlBOB1*‐silenced plants (Figs 6 and 7). For example, a 38‐fold decrease vs. a 14‐fold reduction in bacterial populations were observed in *SlBOBs*‐co‐silenced and *SlBOB1*‐silenced plants (Figs 6 and 7), respectively, as compared with those in *GUS*‐silenced plants, at 3 dpi. This increased level of resistance in *SlBOBs*‐co‐silenced plants over that in *SlBOB1*‐silenced plants indicates a function of SlBOB2 and SlBOB3 in immunity against *Pst* DC3000. Despite the existence of interaction in planta, SlSAP3 and SlBOB1/SlBOB2/SlBOB3 play opposite roles in tomato immunity against *Pst* DC3000. SlSAP3 functions as a positive regulator while SlBOB1 and SlBOB2/SlBOB3 act as negative regulators. Arabidopsis AtBOB1 was previously found to be required for organismal thermotolerance (Perez *et al*., 2009). Thus, it seems likely that plant BOB proteins have diverse functions in biotic and abiotic stress responses. Notably, silencing of either *SlSAP3* or *SlBOB1* affected the *Pst* DC3000‐induced SA signalling regulatory and defence genes but not the JA/ET signalling genes (Figs 3 and 6). These observations suggest that a same defence signalling pathway is associated with the functions of SlSAP3 and SlBOB1, although they play opposite roles in tomato immunity against *Pst* DC3000.

In summary, we demonstrated that SlSAP3 acts as a positive regulator of immunity against *Pst* DC3000 in tomato through the SA signalling. We also found that SlSAP3 interacted with members of SlBOB family, which act as negative regulators of tomato immunity against *Pst* DC3000. As SlSAP3 does not possess ubiquitin E3 ligase activity *in vitro*, SlSAP3 may exert its function in immunity by interacting with other proteins associated with UPS. However, the mechanism by which the interaction of SlSAP3‐SlBOBs regulates immunity is an open question to be investigated further. Because both SAP and BOB proteins seem to be associated with UPS via interaction with UPS subunits or components (Choi *et al*., 2012; Farmer *et al*., 2010; Fu *et al*., 2010; Gunsalus *et al*., 2005; Kang *et al*., 2017; Lloret *et al*., 2017; Saeki, 2017), the interaction of SlSAP3‐SlBOBs *in planta* might initiate an event that results in the degradation of one or both of them and of other unknown targeting proteins upon pathogen infection. Further characterization of SlSAP3 and SlBOB targets of SlSAP3 will be helpful to understand the biochemical mechanism of SlSAP3‐SlBOBs complex in tomato immunity.

## Experimental Procedures

### Plant growth, treatment and disease assays

Tomato (*Solanum lycopersicum* L.) cv. Ailsa Craig was used for all experiments. Plants were grown in a mixture of perlite: vermiculite: plant ash (1 : 6 : 2) in a growth room under fluorescent light (200 µmol/m^2^/s) at 22 °C–24 °C with 60% relative humidity and a 14 h light/10 h dark cycle. Pathogen inoculation, disease assays and measurement of *in planta* bacterial growth were performed basically according to previously described protocols (Li *et al*., 2014).

### **Virus‐induced gene silencing** (**VIGS) assays**

VIGS fragments of 13 *SlSAP* genes (Solanke *et al*., 2009) and three *SlBOB* genes were amplified using gene‐specific primers and cloned into the pTRV2 vector (Liu *et al*., 2002), yielding plasmids pTRV‐*SlSAPs* or pTRV‐*BOBs*. Sequence information for the VIGS fragments is listed in Table S1. In the case of co‐silencing of *SlBOBs*, A 278 bp fragment, designated as SlBOBa that corresponds to the conserved regions in open reading frames (ORFs) of the *SlBOB* genes, was cloned into the pTRV2 vector, yielding pTRV‐*SlBOBa*. Standard VIGS procedure was applied to 10‐day‐old tomato seedlings (Li *et al*., 2014; Liu *et al*., 2002). Silencing efficiency and specificity were analysed by qRT‐PCR at 3 weeks after VIGS manipulation. The primers used are listed in Table S3 (see Supporting Information).

### Generation of *SlSAP3*‐OE transgenic lines

The coding sequence of *SlSAP3* was amplified with primers SlSAP3‐OE‐HA‐F and SlSAP3‐OE‐HA‐R (Table S3, see Supporting Information) and cloned into plant transformation vector pFGC1008‐HA at *Asc*I/*Kpn*I sites under the control of the CaMV 35S promoter. The resulting construct was introduced into tomato cv. Ailsa Craig by *Agrobacterium tumefaciens*‐mediated transformation (Abuqamar *et al*., 2008; Howe *et al*., 1996). Transformants were selected based on their resistance to Hygromycin B. Homozygous T2 or T3 transgenic plants were used for phenotypic and molecular characterization.

### Reactive oxygen species (ROS) assays

ROS assays were carried out as described previously (Shang‐Guan *et al*., 2018). Briefly, leaf discs (0.2 cm^2^) were incubated overnight in a 96‐well plate with water, and 200 mM luminol (Sigma‐Aldrich, Saint Louis, MO, USA), 20 mg/mL horseradish peroxidase (Sigma‐Aldrich, Saint Louis, MO, USA), or 100 nM flg22 (GenScript, Nanjing, China) were then added. Chemiluminescent signal was recorded at a 2 min interval over 30 min using a Synergy HT plate reader (Biotek Instruments, Inc., Winooski, VT, USA).

### Purification of recombinant GST‐SlSAP3 protein and ubiquitin E3 ligase activity assay

The coding sequence of *SlSAP3* was amplified with a pair of primers (Table S3, see Supporting Information) and cloned into pGEX‐4T‐3 vector at *Bam*HI/*Xho*I sites. The resulting plasmid was introduced into the *E. coli* strain Rosetta DE3 and expression of GST‐SlSAP3 fusion was induced by adding of 1 mM isopropyl‐D‐thiogalactoside (IPTG) at 20 °C overnight. Recombinant GST‐SlSAP3 fusion protein was purified using the Bug‐Buster GST‐Bind purification kit according to the manufacturer's protocol (Novagen, Darmstadt, Germany). A GST tag sample was also purified from *E. coli* cells with the same protocol. Protein concentration was determined using Bio‐Rad protein assay kit (Bio‐Rad, CA, USA) following the recommended method. Ubiquitination assays were performed as described previously (Zhao *et al*., 2012). Briefly, reactions (30 μL) contained 5 μg ubiquitin (Boston Biochem, Cambridge, MA, USA), 110 ng E1 (Merck Millipore, Darmstadt, Germany), 100 ng human recombinant UbcH2 (Abcam, Cambridge, UK), and purified 4 μg GST‐SlSAP3 in buffer (20 mM MOPS, pH 7.2, 100 mM KCl, 5 mM MgCl_2_, 5 mM ATP and 10 mM DTT) and were incubated at 30 °C for 3 h. Reactions were stopped by adding SDS sample buffer and analysed by SDS‐PAGE. followed by immunoblotting using anti‐ubiquitin antibody (CalBiochem, La Jolla, CA, USA). Chemiluminescence signal was detected with SuperSignal West Pico PLUS Chemiluminescent Substrate kit (Thermo Fisher Scientific, Waltham, MA, USA) according to the manufacturer's recommendations.

### Y2H screening and verification of SlSAP3 interactors

A tomato Y2H library constructed with cDNAs prepared from *Pst *DC3000‐infected leaves was screened using SlSAP3 as a bait. The co‐transformed yeast cells were selected on QDO (SD/‐Ade/‐His/‐Leu/‐Trp) medium and the survivals were further screened on QDO medium containing 40 μg/mL X‐α‐Gal and 125 ng/mL Aureobasidin A. The AD plasmids were rescued from putative positive clones and sequenced. For determining the domains responsible for interaction in SlSAP3, the coding sequence of *SlSAP3* and its deletion mutants (SlSAP3‐A20, SlSAP3‐AN1 and SlSAP3ΔA20ΔAN1) were PCR amplified with gene‐specific primers (Table S3, see Supporting Information) and cloned in‐frame into pGBKT7 plasmids. Similarly, the coding sequences of *SlBOBs* were amplified with gene‐specific primers (Table S3, see Supporting Information) and cloned in‐frame into pGADT7 plasmids. Combined pairs of recombinant pGBKT7 plasmids containing SlSAP3 or its deletions mutants and pGADT7 plasmids harbouring SlBOBs were co‐transformed into yeast cells and the interaction activity was examined by plating yeast cells on DDO medium and QDO medium containing 40 μg/mL X‐α‐Gal and 125 ng/mL Aureobasidin A.

### **Bimolecular fluorescence complementation** (**BiFC) assays**

BiFC assays for determining the interaction between SlSAP3 and SlBOBs were performed as described previously (Yang *et al*., 2007). The coding sequence of *SlSAP3* was amplified with gene‐specific primers and cloned into p2YC at *Pac*I‐*Asc*I sites, yielding plasmid p2YC‐SlSAP3 that codes for a fusion with the C‐terminal fragment of YFP. Similarly, the coding sequences of *SlBOB1 and SlBOB2 *were amplified with gene‐specific primers and cloned into p2YN at the *PacI*‐*Asc*I sites, yielding plasmids p2YN‐SlBOB1 and p2YN‐SlBOB2 that code for fusions with the N‐terminal fragment of YFP. BiFC experiments were performed in leaves of 2‐week‐old *N. benthamiana* plants expressing a known nucleus‐localized marker protein RFP‐H2B (Chakrabarty *et al*., 2007) as described previously (Yang *et al*., 2007). YFP and red florescent protein (RFP) fluorescence were observed and photographed by a Zeiss LSM780 confocal laser scanning microscope (Zeiss, Oberkochen, Germany) 48 h after agroinfiltration. The primers used for BiFC assays are listed in Table S3 (see Supporting Information).

### Co‐immunoprecipitation (co‐IP) assays

Co‐IP assays were conducted according to a previously described procedure (Zhu *et al*., 2014). Briefly, the coding sequences of *SlSAP3* and *SlBOB1* were amplified using gene‐specific primers and cloned into pFGC‐eGFP vector with a GFP tag at the N‐terminus, yielding plasmids pFGC‐eGFP‐SlSAP3 and pFGC‐eGFP‐SlBOB1. Similarly, the coding sequence of *SlSAP3* and *SlBOB2* were amplified with a pair of gene‐specific primers and cloned into pFGC1008‐HA, yielding plasmid pFGC1008‐SlSAP3‐HA and pFGC1008‐SlBOB2‐HA. Agrobacteria harbouring pFGC1008‐SlSAP3‐HA and pFGC‐eGFP‐SlBOB1 or harbouring pFGC1008‐SlBOB2‐HA and pFGC‐eGFP‐SlSAP3 were combined and infiltrated into the abaxial air spaces of leaves of 4‐week‐old *N. benthamiana* plants. The agroinfiltrated leaves were collected at 48 h after agroinfiltrations and total proteins were extracted with extraction buffer (50 mM HEPES, pH 7.5, 100 mM NaCl, 5 mM EDTA, 50 mM EGTA, 25 mM NaF, 1mM NaVO_3_, 50mM β‐glycerophosphate, 20% [v/v] glycerol, 1mM PMSF, 0.1% [v/v] Triton X‐100, 1 mM DTT and 1 × protease inhibitor cocktail [Sigma‐Aldrich, Saint Louis, MO, USA]). After centrifugation at 12 000 *g* for 10 min, 1 mL of supernatant was mixed with GFP‐Trap (ChromoTek, Planegg‐Martinsried, Germany) and rotated overnight at 4 °C. After washing four times with extraction buffer, the GFP‐Trap beads were resuspended in 50 µL 2 × SDS sample buffer and boiled for 10 min at 95 °C to dissociate immunoprecipitated protein complex. The dissociated immunoprecipitated proteins were separated on 12% SDS‐PAGE and were detected by immunoblotting with anti‐GFP‐specific antibody (Sigma‐Aldrich, Saint Louis, MO, USA) or anti‐HA‐specific antibody (Sigma‐Aldrich, Saint Louis, MO, USA). The primers used are listed in Table S3 (see Supporting Information).

### Quantitative Reverse Transcription‐Polymerase Chain Reaction (qRT‐PCR) analysis of gene expression

Total RNA was extracted by Trizol reagent (TaKaRa, Dalian, China) according to the manufacturer's instructions. RNA was treated with RNase‐free DNase and then reverse‐transcribed into cDNA using the PrimeScript RT reagent kit (TaKaRa, Dalian, China). The obtained cDNAs were used for gene expression analysis by real‐time qPCR. Each qPCR reaction contained 12.5 µL SYBR Premix Ex Taq (TaKaRa, Dalian, China), 0.1 µg cDNA and 7.5 pmoL of each gene‐specific primer (Table S3, see Supporting Information) in a final volume of 25 µL, and was run in a CFX96 real‐time PCR detection system (Bio‐Rad, Hercules, CA, USA). A tomato *SlActin* gene (Accession No. AB199316) was used as an internal control to normalize the qRT‐PCR data and relative expression levels of genes of interest were calculated using the 2^−ΔΔCt^ method. Three independent biological samples were performed.

## Conflict of Interest

The authors declare no conflicts of interest.

## Supporting information


**Fig. S1** Silencing efficiency and specificity for target genes. (A) Silencing efficiency of each of the *SlSAP* genes in corresponding virus induced gene silencing (VIGS) infiltrated plants. (B) Silencing specificity in pTRV *SlSAP3* infiltrated plants. (C) Silencing efficiency and specificity in pTRV SlBOB1 infiltrated plants. Two week old tomato seedlings were infiltrated with agrobacteria carrying pTRV *SlSAPs*, pTRV *SlBOB1* or pTRV *GUS* constructs and leaf samples were collected at 4 weeks after agroinfiltration. Transcript levels of each of the *SlSAP* and *SlBOB1* genes in corresponding pTRV *SlSAP*  or pTRV *SlBOB1* infiltrated and pTRV *GUS* infiltrated plants were analysed by quantitative Reverse Transcription Polymerase Chain Reaction (qRT PCR). *SlActin* was used as an internal reference gene and relative expression was shown as folds of the transcript value of the *SlActin* gene. Data presented are the means ± standard errors (SE) from three experiments with independent biological samples. Statistical significance compared with pTRV *GUS* was determined by Student's *t*‐tests: *P < 0.05. All experiments were repeated three times with similar results.

**Fig. S2** Western blot analysis to detect the expression of bimolecular fluorescence complementation (BiFC) constructs shown in Figs 5B and 7B. Immunoblot analysis of p2YN HA SlBOB1, p2YN HA SlBOB2 and p2YC HA SlSAP3 fusion proteins in* Nicotiana benthamiana* leaves at 48 h after agroinfiltration. A HA specific antibody was used for detection of HA fusion protein. Equal loading of total proteins was examined by Ponceau staining.

**Fig. S3** Subcellular localization of SlSAP3 and SlBOBs. Agrobacteria carrying pFGC eGFP *SlSAP3*, pFGC eGFP *SlBOBs* or pFGC eGFP empty vector were infiltrated into leaves of *Nicotiania benthamiana *plants expressing a red nucleus marker protein RFP H2B and leaf samples were collected at 48 h after infiltration for observation under a confocal laser scanning microscope. Images were taken in dark field for green fluorescence (left) and red fluorescence (middle left), white field for cell morphology (middle right) and in combination (right), respectively.

**Fig. S4** Sequence alignment and phylogenetic tree analysis of SlBOBs. (A) Alignment of SlBOBs with Arabidopsis AtBOBs. The conserved C terminal NudC domain regions is underlined. Numbers on the right indicate amino acid positions of the BOB proteins. **(B)** Phylogenetic tree analysis of SlBOBs with other plant BOBs. Phylogenetic tree was constructed by Neighbour joining method using MEGA7 programme. Plant BOBs used and their GenBank accessions are as follows: *Arabidopsis thaliana* AtBOB1 (NP_200152), AtBOB2 (NP_194518), *Oryza sativa* OsBOB1 (XP_015640993), *Solanum lycopersicum* SlBOB1 (XP_004234959), SlBOB2 (XP_004233975), SlBOB3 (XP_025887281), *Nicotiana tabacum* NtBOB1 (XP_016451285), NtBOB2 (XP_016468156), *Brassica rapa* BrBOB1 (XP_009132480), BrBOB2 (XP_009108672) and *Glycine max* GmBOB1 (XP_003526709). Bootstrap values from 1000 replicates are indicated at each node. Bar represents the number of amino acid differences per site.

**Fig. S5** Phylogenetic tree analysis of SlSAP3 with other reported plant stress associated proteins (SAPs). Phylogenetic tree was constructed by Neighbour joining method using MEGA7 programme. SAPs involved in plant immunity are indicated by red arrows. Plant SAPs used and their GenBank accessions are as follows: *Arabidopsis thaliana *AtSAP5 (NP_566429), AtSAP9 (NP_194013), AtSAP10 (NP_194268), AtSAP12 (NP_189461), AtSAP13 (NP_191307), *Aeluropus littoralis* AlSAP (ABK90631), *Festuca arundinacea* FaZnF (AEZ53300), *Leymus chinensis* LcSAP (CD808976) *Lobularia maritima* LmSAP (AUN86611), *Malus domestica* MdSAP15 (XP_008375158), *Medicago truncatula* MtSAP1 (XP_024626996), *Musa acuminata *MusaSAP1 (XP_009411822), *Oryza sativa *OsSAP1 (XP_015651267), OsSAP7 (XP_015633143), OsSAP8 (XP_015643189), OsSAP9 (XP_015647896), OsSAP11 (XP_015651039), OsSAP16 (XP_015644892), *Phalaenopsis aphrodite* Pha13 (PATC148746), *Prunus persica* PpSAP1 (XP_007218502), *Saccharum officinarum* ShSAP1 (ACT53874), *Solanum lycopersicum* SlSAP3 (ACM68440), *Sorghum bicolor* SbSAP14 (XP_002466323) and *Zea mays* ZmAN13 (AQL04999). Bootstrap values from 1000 replicates are indicated at each node. Bar represents the number of amino acid differences per site.

**Table S1** Sequence of the virus‐induced gene silencing (VIGS) fragments for *SlSAP* and *SlBOB* genes.

**Table S2** Putative SlSAP3 interactors identified by Y2H screening.

**Table S3** Primers used in this study for different purposes.

**Table S4** CDS and amino acid sequences of the *SlSAP* and *SlBOB* genes.
