# Supplementary figures and images for: Tomato SlSAP3, a member of the stress‐associated protein family, is a positive regulator of immunity against Pseudomonas syringae pv. tomato DC3000
Source: Mol Plant Pathol. 2019 Mar 25;20(6):815–30. doi: 10.1111/mpp.12793 (PMC6637894; doi:10.1111/mpp.12793)

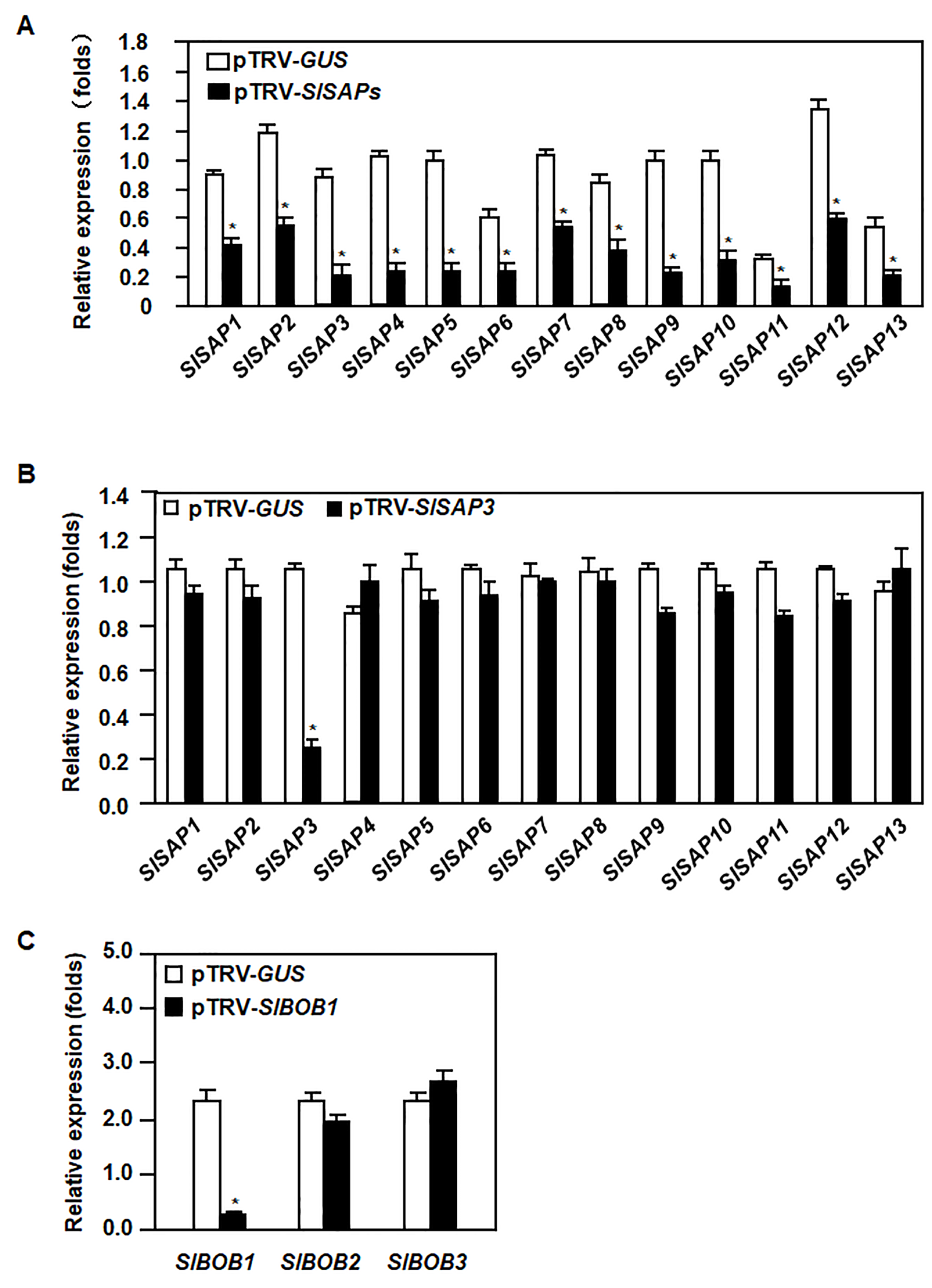

Supplement: Supplementary file 1 — Fig. S1 Silencing efficiency and specificity for target genes. (A) Silencing efficiency of each of the SlSAP genes in corresponding virus induced gene silencing (VIGS) infiltrated plants. (B) Silencing specificity in pTRV SlSAP3 infiltrated plants. (C) Silencing efficiency and specificity in pTRV SlBOB1 infiltrated plants. Two week old tomato seedlings were infiltrated with agrobacteria carrying pTRV SlSAPs, pTRV SlBOB1 or pTRV GUS constructs and leaf samples were collected at 4 weeks after agroinfiltration. Transcript levels of each of the SlSAP and SlBOB1 genes in corresponding pTRV SlSAP or pTRV SlBOB1 infiltrated and pTRV GUS infiltrated plants were analysed by quantitative Reverse Transcription Polymerase Chain Reaction (qRT PCR). SlActin was used as an internal reference gene and relative expression was shown as folds of the transcript value of the SlActin gene. Data presented are the means ± standard errors (SE) from three experiments with independent biological samples. Statistical significance compared with pTRV GUS was determined by Student's t‐tests: *P < 0.05. All experiments were repeated three times with similar results. [file MPP-20-815-s001.tif]

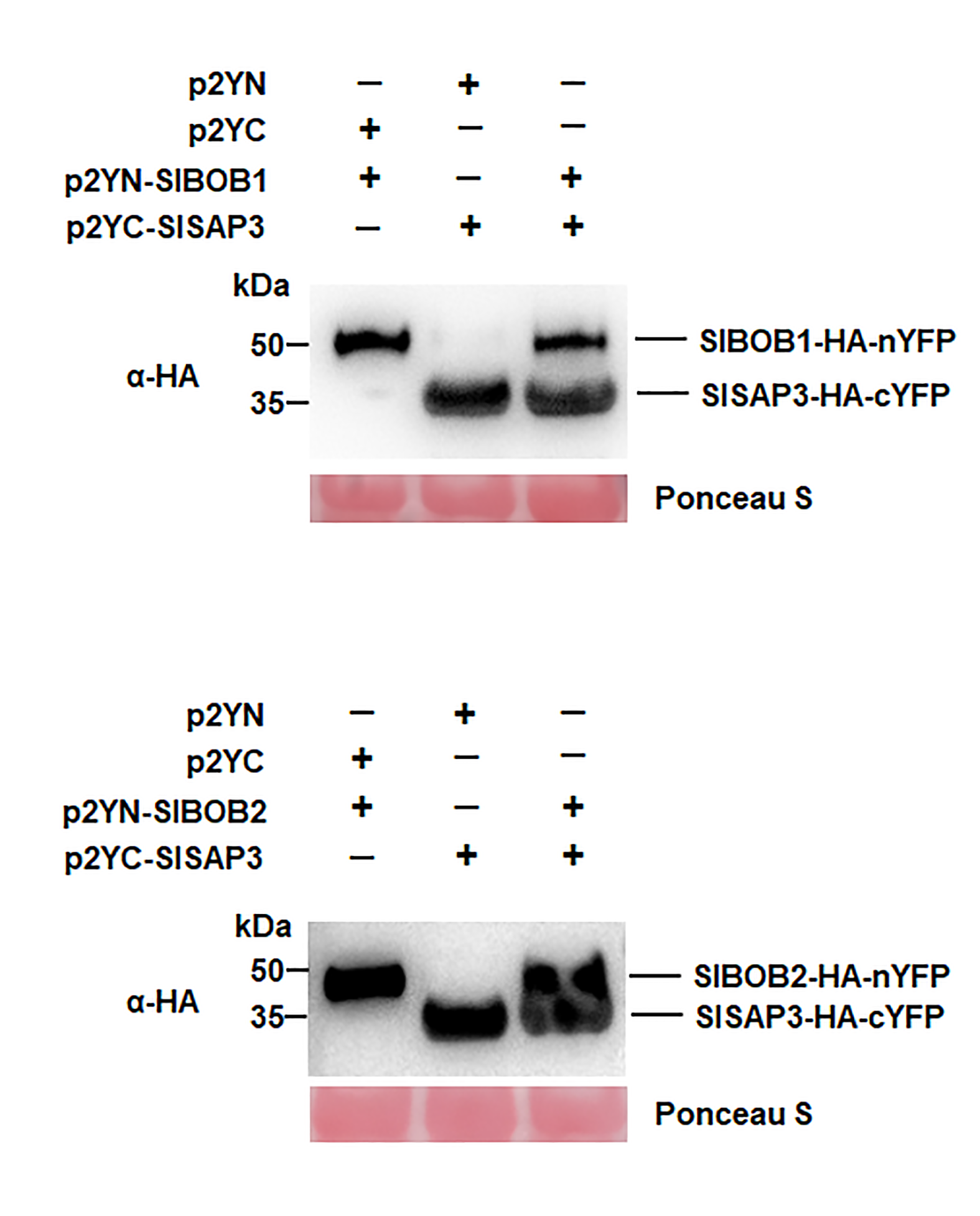

Supplement: Supplementary file 2 — Fig. S2 Western blot analysis to detect the expression of bimolecular fluorescence complementation (BiFC) constructs shown in Figs 5B and 7B. Immunoblot analysis of p2YN HA SlBOB1, p2YN HA SlBOB2 and p2YC HA SlSAP3 fusion proteins in Nicotiana benthamiana leaves at 48 h after agroinfiltration. A HA specific antibody was used for detection of HA fusion protein. Equal loading of total proteins was examined by Ponceau staining. [file MPP-20-815-s002.tif]

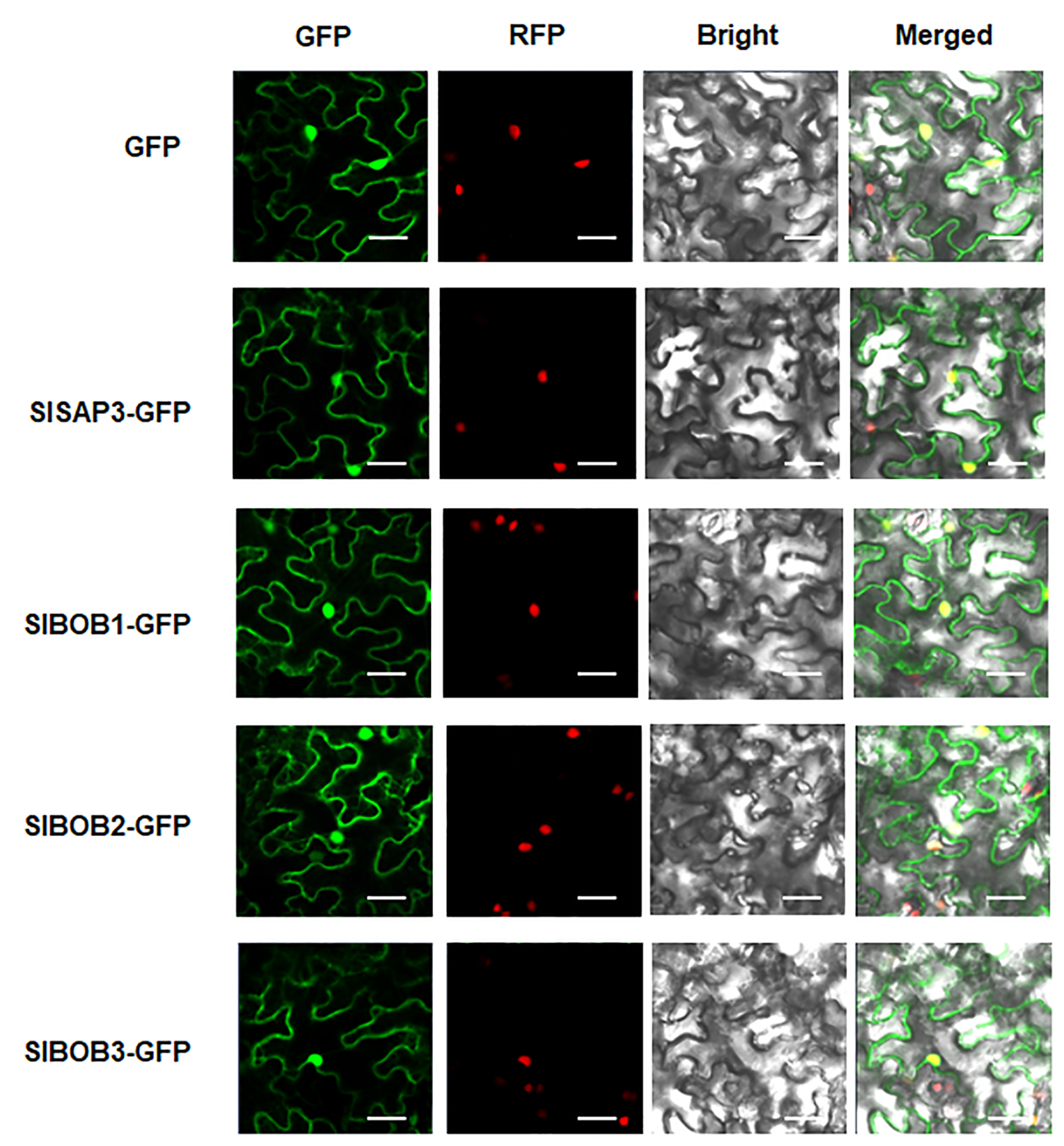

Supplement: Supplementary file 3 — Fig. S3 Subcellular localization of SlSAP3 and SlBOBs. Agrobacteria carrying pFGC eGFP SlSAP3, pFGC eGFP SlBOBs or pFGC eGFP empty vector were infiltrated into leaves of Nicotiania benthamiana plants expressing a red nucleus marker protein RFP H2B and leaf samples were collected at 48 h after infiltration for observation under a confocal laser scanning microscope. Images were taken in dark field for green fluorescence (left) and red fluorescence (middle left), white field for cell morphology (middle right) and in combination (right), respectively. [file MPP-20-815-s003.tif]

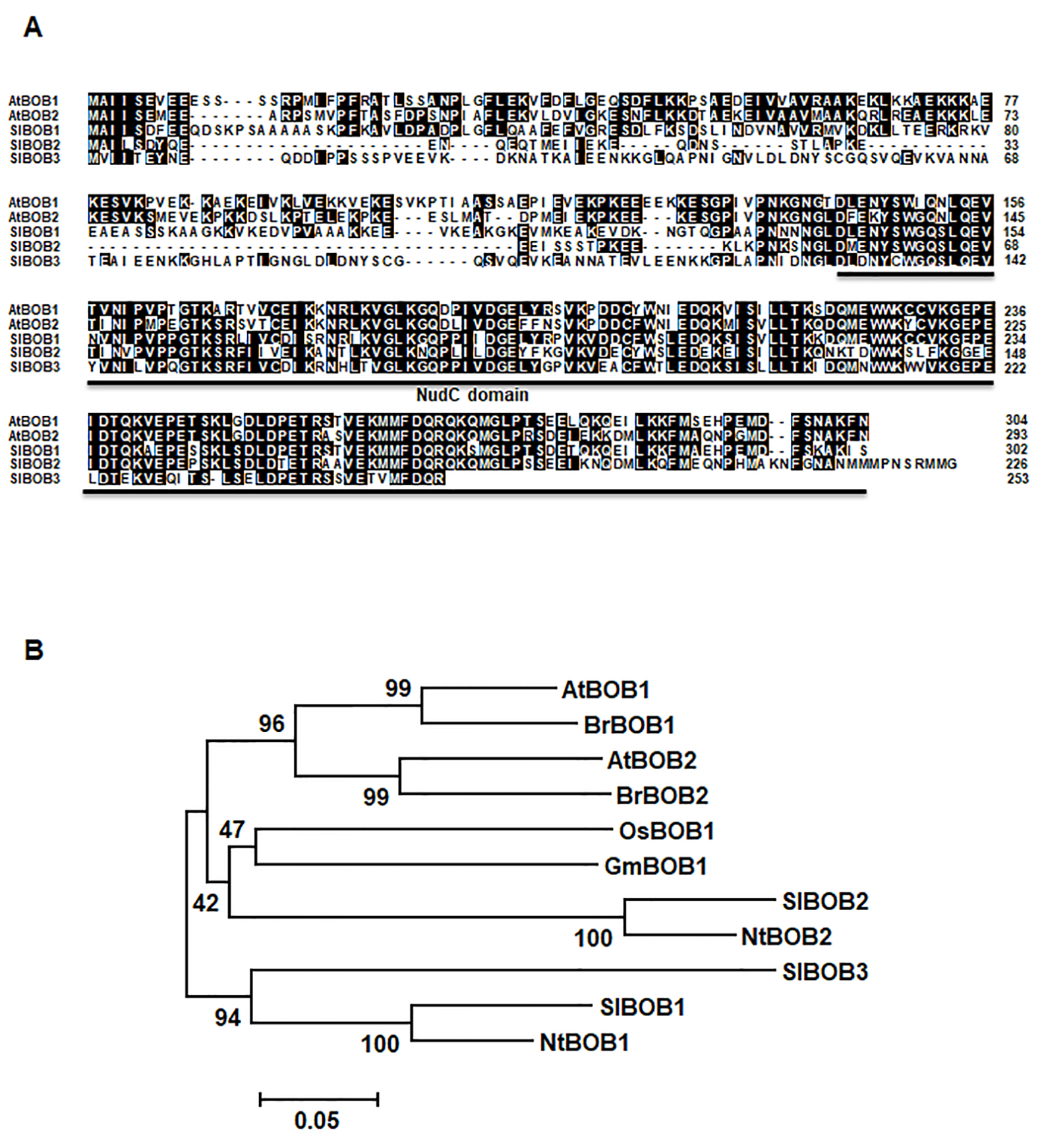

Supplement: Supplementary file 4 — Fig. S4 Sequence alignment and phylogenetic tree analysis of SlBOBs. (A) Alignment of SlBOBs with Arabidopsis AtBOBs. The conserved C terminal NudC domain regions is underlined. Numbers on the right indicate amino acid positions of the BOB proteins. (B) Phylogenetic tree analysis of SlBOBs with other plant BOBs. Phylogenetic tree was constructed by Neighbour joining method using MEGA7 programme. Plant BOBs used and their GenBank accessions are as follows: Arabidopsis thaliana AtBOB1 (NP_200152), AtBOB2 (NP_194518), Oryza sativa OsBOB1 (XP_015640993), Solanum lycopersicum SlBOB1 (XP_004234959), SlBOB2 (XP_004233975), SlBOB3 (XP_025887281), Nicotiana tabacum NtBOB1 (XP_016451285), NtBOB2 (XP_016468156), Brassica rapa BrBOB1 (XP_009132480), BrBOB2 (XP_009108672) and Glycine max GmBOB1 (XP_003526709). Bootstrap values from 1000 replicates are indicated at each node. Bar represents the number of amino acid differences per site. [file MPP-20-815-s004.tif]

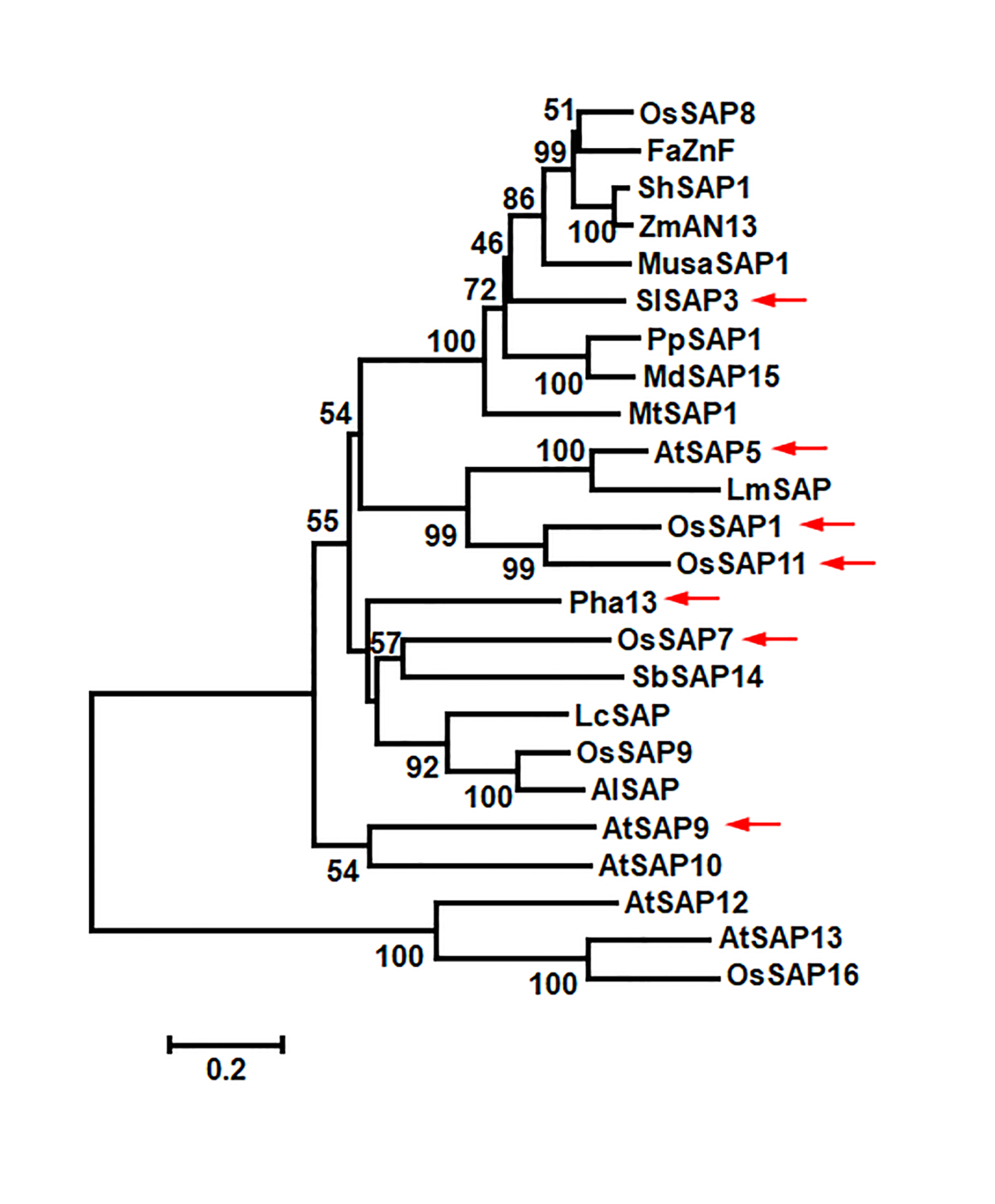

Supplement: Supplementary file 5 — Fig. S5 Phylogenetic tree analysis of SlSAP3 with other reported plant stress associated proteins (SAPs). Phylogenetic tree was constructed by Neighbour joining method using MEGA7 programme. SAPs involved in plant immunity are indicated by red arrows. Plant SAPs used and their GenBank accessions are as follows: Arabidopsis thaliana AtSAP5 (NP_566429), AtSAP9 (NP_194013), AtSAP10 (NP_194268), AtSAP12 (NP_189461), AtSAP13 (NP_191307), Aeluropus littoralis AlSAP (ABK90631), Festuca arundinacea FaZnF (AEZ53300), Leymus chinensis LcSAP (CD808976) Lobularia maritima LmSAP (AUN86611), Malus domestica MdSAP15 (XP_008375158), Medicago truncatula MtSAP1 (XP_024626996), Musa acuminata MusaSAP1 (XP_009411822), Oryza sativa OsSAP1 (XP_015651267), OsSAP7 (XP_015633143), OsSAP8 (XP_015643189), OsSAP9 (XP_015647896), OsSAP11 (XP_015651039), OsSAP16 (XP_015644892), Phalaenopsis aphrodite Pha13 (PATC148746), Prunus persica PpSAP1 (XP_007218502), Saccharum officinarum ShSAP1 (ACT53874), Solanum lycopersicum SlSAP3 (ACM68440), Sorghum bicolor SbSAP14 (XP_002466323) and Zea mays ZmAN13 (AQL04999). Bootstrap values from 1000 replicates are indicated at each node. Bar represents the number of amino acid differences per site. [file MPP-20-815-s005.tif]
