# Supplementary material for: Tomato SlSAP3, a member of the stress‐associated protein family, is a positive regulator of immunity against Pseudomonas syringae pv. tomato DC3000
Source: Mol Plant Pathol. 2019 Mar 25;20(6):815–30. doi: 10.1111/mpp.12793 (PMC6637894; doi:10.1111/mpp.12793)
Supplement: Supplementary file 6 — Table S1 Sequence of the virus‐induced gene silencing (VIGS) fragments for SlSAP and SlBOB genes. [file MPP-20-815-s006.docx]

**Supplementary Table S1:** **Sequence of the VIGS fragments for *SlSAP* and *SlBOB* genes**

**SlSAP1-vigs:** 343 bp

AGCAGGCAACCGAATTTTGTCATAATTTTAAAATTTTACACACACAAAAAGAAAAATGGCTCAGAGAACGGAGAAAGAAGAGACGGAGTTCAAGGCCGTACCTGAAACGATAACGCTTTGCATCAACAATTGTGGAGTTACAGGAAATCCAGCCACAAACAATATGTGTCAAAAGTGCTTCAACGCCACCACCGCGGCTACCTCAACTTCATCCAGCTCGCCGACGGGGACGTCGGTGACGATTCCTCACAATTTCGCTGAAAAATTGGTCAGATCTGAAAAATCGGCGAGATTCAGCTCGTTGAGGTCGTCGCCGGACAGGAAGTCTGATCTGGACAGGATG

**SlSAP2-vigs:** 354 bp

AGCAACAATATGGAGCATGATGAGACAGGATGTCAACCCCATCCAGAAGGCCCTATTTTGTGCATTAACAATTGTGGGTTTTTTGGAAGTGCTGCAAATATGAATATGTGCTCCAAGTGTTATAAGGACGTGATTCTGAAACAAGAACAAGAAAAGCTTGCAGCATCGTCAATTGAAAACTTTGTGAATGGGTCAACAAGTCAGAAGGGGCCTGTCATTGTTGGCTCTGTGGATGTGCAACCTGCCCTGCTAGAATCAAAATCTGTGGTTTTGTCATCACCTCCATCTTCAAGTTCTGGTGAGGCTGCTGAATTGATGGCCAAGGAGGGTCCTAGCCGATGCAGCACTTGCAAG

**SlSAP3-vigs:** 326 bp

TTCAAATGTCGCTGTGGTAACCTTTATTGTGGAGCACACCGCTACTCAGATAAACATGACTGCCTGTTTGATTACCGCTCTGCTGGCCAGGATGCAATAGCGAAGGCCAACCCTGTTGTTAAGGCAGAGAAGCTTGACAAGATCTAGAAGGGGAGGGAGTATTTGGTGATTTGATTTGGTCTCGAAAAGGGTCCTTCTTTGATTGGCAAGATATATCTTGAGATCTTATTAATCCTGGGGGACGCAGAAGAAAAAACGATATGTGCATGTTGAAGAATTATTAGGAGAATTTGTGTGTTTGCTCGATTGTCAGGTTTTTCATTTTG

**SlSAP4-vigs:** 316 bp

GCTCAGCTCATCGCTACTCAGACAAACATGAGTGTCCATATGATTATCGCAAGGCTGGTCAAGATGCTATTGCGAAAGCCAACCCAGTTGTTAAGGCTGAAAAGCTTGACAAAATATGAAGGAACATCCTGAGTTTATGAGCTGCAGTATTATTGAAGACGTCTTCCATACCCATCTGGTGACTTCTACTGTTTGGTGCCTAGGATCTCGAGGGGGTCGAGAGAGAGAATCATAAGGCATCTTTACAACTTGAGGGGTCGTAATGTTGGTATCAAGTAAGAAATGTTGTGGTTTCCATCTTTAGTGCTTGTTTTGT

**SlSAP5-vigs:** 351 bp

TTATGGTGTGGCAAGTGACAGGTTAACTGCTGCACAAAATGGAGTCTTCCAAAGAAACAGGCTGTCAAGCTCCCGAAGGTCCCATCCTATGCATCAACAACTGCGGGTTCTTTGGTAGTGCAGCCACTATGAATATGTGTTCCAAGTGTCACAAGGACATGATACTGAAGCAGGAACAAGCTAAATTTGCAGCAACATCAATTGAAAACATAGTAAATGGAAACTCAAGCAGCAATGGCAAAGAGCCTATTGCGACTGGTGCAATCAATGTTCAACCGGGATCAGCAGACTTGAAGGTTATCTCTACAGAAGCATCTTCTGATTTATCTTCAGGTCCAAGTTCAGAGGTGA

**SlSAP6-vigs:** 337 bp

TAATACAGAAGAAAATCATGGGTTCTGAAGGCAACAAGTTTAACGATGGAACAAGCTTCCCGCCGGCGGATCCGATTCTCTGTTCAAACGGTTGCGGATTTTTTGGTACGGCGGCGACGAATGGACTTTGTTCAAAGTGTTACCGTGACTTTAAGATGAAGGAAGATCATGCTGCGATGGCTAAAGTAGCGATGGAGAAACTTGTTATATCTAGACCTCAAATTGAATCGATCGGAAAAGTTGATTTCTGTTCGTCGACTACATCGACGGCTGCGGAGAGGCCGGTTGTTGAGGCGGCGACGGCGGAGATCGGCGGGAGTCAGCCGAATCGGTGTTTG

**SlSAP7-vigs:** 360 bp

TTCGTTGAGTTAAGAATCACAGTTTGTTTGAAAGAAAAAAAGATGGCGGAAGAACAAAGGATGCAAGAAGGAGGAGGACATAGGCTATGTGCTAATAATTGTGGTTTCTTTGGTAGCCCAACAACTTTGAACCTTTGTTCCAAATGCTACAAGGATCATTGCATGAAAGAACAACAATCGCGAACCGCTCAGCTCGCAATGGAAAAGACTCGTCCCCAACAACAACAACAACAACAATCTGAATCAACGTCTACGTACATACCATGTACAAAGCCGTTACCAATTCTTGAAGTCTCACAACCACGAGAGACAGAGATTGCAACTAGGGCTCCTCAGGTGCAGTCAGATACTGCAGCTGAG

**SlSAP8-vigs:** 372 bp

TCCTTTCTCCCTATTTTCAAGATTCTTCATTCACCCTTTTTTTTCCATTTTTCTCTCCATCTTTTCTTCCATTTTTCAACACCCTTTTGCTTTAATTACAAAACCCATAATTTATTTTCATCAAATATTCAATCAAAAAAAATTATTTCTATGGCGGAAGAACATGAATTTCAATCCCAAGAAGGTGGACGTCACCAATTATGTGCAAATAATTGTGGTTTTTTTGGCAACTCTACAACCGAAAATTATTGTTCAAAATGTTATCGCGATATCGAAAAACAAAAATCCGATGCAAAATCAATCGATTCTCTTTTTTCTCCGATAAAAAAGGTTTCGGAAAAAAAGATAATCGAGCCGATTGTTTTGACGACG

**SlSAP9-vigs:** 364 bp

TGATATCGAAGAGAAATCGATATAATTATGGCGGAAGAACATGGATTTGAAGCACCAGAAGGACATATATTATGCGCTAATAACTGTGGTTTCTTTGGTAGCCCAACAACGCAAAATTTCTGCTCCAAATGTTACAATGAAGTTTACATAAAAGGGGGACAACAAAAACCTATTGATTCCCTTTTTCCTCCTTCGCAGCTGCCGATTCCATCAACCTCATCGATACTGGTGCTGCAGGAATCGACAGCTGCGGAGGAAGAACCTGAGGTTGTGACCGCGGCTGTAACAGTCGCGGTCCAACCGATTTCTGCACAGCCGAACAGATGTTCGGCCTGCAGGAAGAAGGTGGGATTGACAGGGTTCA

**SlSAP10-vigs:** 378 bp

GCCAAACACTTCCCAACACCCCAAATTCCCATTTCATTTCCATTCTCTCTCTCTAGAAATCTCTCCATCATGGCGGCGCAGAAGAGAGAGAAAGAAGAAACCGAGCTGAAGGTGCCGGAATCCATCCCTCTATGCTCTCCAACTCTACCGGTACCTTCACCATCGCCTCCTTCTACGACGACGCACCTCTCAGTTGCTGTTATCTCAGATCTGAAGCGTTCTGATAGATCGTCGACGGAGAGTATAGATCTGAAGGTTTCTAGTATGGATGATCAATCGAGATCTACATCAGCTGCATCGCCGGAAAGTATGGATCTGGTTGGTAGAAAAACAGGGGTTAAGAGGCAAAGAGAAGCGAACCGATGTTCTGGTATGGGT

**SlSAP11-vigs:** 352 bp

CACGAAAGAAGAAAATGGAAGGAGGAACAGAAGCCTATCCAGATTTAGGTAGACATTGCCAACTATCTGATTGCCATCAACTCGATTTTCTCCCTTTTACCTGCCATGCCTGTTTAAAGGTATTTTGTGTGGAACATAGATCATGCAAGTCTCATGAATGCCCAAAATCTGACTTTAACAGCCGAATCGTTTTGGTTTGCGAAATTTGTTCTATGTCCATGGAAACTACCGGCTGTAAAGTTGAAGACCACAAAGCAATATTACAAAAACACGAGGAATCTGGGGATTGTGACCCTAAGAAGAAGAAGAAGAAACCTACCTGTCCTGTCAAAAGATGCAAGGGGATTTTGAC

**SlSAP12-vigs:** 359 bp

TTTGCTTGAAGTTGTTGAGTGATAATTTCAATGGGAACGCCAGAGTTCCCAAATCTTGGAAAACATTGCTTTGTAGATGACTGCAGGCAGATTGATTTCTTGCCTTTTACCTGCGATTGTTGTCACCAGGTCTTTTGTCTAGAGCATCGGAGCTATAATAGACACCACTGTCCGACAGCGAACAATAATGATGTTACTGTGGTTGTTTGCCCACTCTGTGCAAAAGGAGTACACCTTATTCCTGACGAAGACCCAAATATAACTTGGGAATCACATGTAAACACAGATTGTGATCCATCAAACTACGAAAAAGCCACAAAGAAAAGAAAATGTCCCGTACCTGGCTGCAGAGAGTTCTT

**SlSAP13-vigs:** 328 bp

GTAGAAAAGTCCTCATTTGCCAGGCAACTTAGGACAGACAACGCTTTGAATCTTTCTCCATTTCTCCAATTCTAGCGTTGTCTATCCTATTGTCTCCTCAAGAATCTTCAAAGAAACCCCATTACCCTAATTTTCAAATTCAATTCTTTTTCTTGTTGTTGTTTACAAAGTTATCAATTCAATGGGTACACCAGAATTCCCAAATCTTGGGAAGCATTGTTCTGTTGAGGATTGTAGGCAGATTGATTTCTTGCCTTTTACCTGTGATTGCTGTTTCAAGGTGTATTGTTTAGATCATCGAAGCTATATTAGACATCAGTGTCCAACG

**SlBOB1-vigs:** 305 bp

GCCGTGCTTGATCCTGCTGATCCTCTAGGGTTTCTTCAAGCCGCGTTCGAGTTCGTCGGACGGGAATCTGATCTTTTCAAGAGTGATTCATTGATTAATGATGTCAATGCTGTTGTTCGTATGGTGAAAGACAAGCTGTTGACTGAGGAGCGCAAGAGGAAAGTGGAAGCAGAAGCATCAAGCTCAAAGGCGGCGGGAAAGAAGGTCAAGGAGGATGTTCCAGTTGCTGCTGCAAAGAAGGAAGAGGTTAAAGAGGCAAAAGGGAAAGAAGTTATGAAGGAGGCTAAGGAGGTGGACAAGAATGG

**SlBOBa-vigs:** 278 bp

ATGGTGATCATTACCGAATACAATGAGCAAGATGACATACCACCATCGTCCTCACCTGTAGAGGAGGTTAAGGACAAGAACGCTACTAAGGCCATTGAAGAGAACAAGAAGGGGCTTCAAGTGTTACAGAGCAACCTTGTTTGTTTCGCATCACCAGTAGCTTATTCTGCTGATATGTACACAGCTCCCAACATAGGCAATGTCCTTGATCTGGACAACTACTCTTGCGGTCAATCCGTACAGGAGGTTAAGGTGGCCAATAATGCTACTGAGGCAAT
