# Supplementary material for: Tomato SlSAP3, a member of the stress‐associated protein family, is a positive regulator of immunity against Pseudomonas syringae pv. tomato DC3000
Source: Mol Plant Pathol. 2019 Mar 25;20(6):815–30. doi: 10.1111/mpp.12793 (PMC6637894; doi:10.1111/mpp.12793)
Supplement: Supplementary file 7 — Table S2 Putative SlSAP3 interactors identified by Y2H screening. [file MPP-20-815-s007.docx]

**Supplementary Table S2: Putative SlSAP3 interactors identified by Y2H screening**

| **Protein Name** | **Gene ID** | **Clone Number** |
| --- | --- | --- |
| **ubiquitin** | Solyc01g056940 | 1, 3, 4, 5, 7, 9, 17, 20, 23, 24 |
| **Ubiquitin-ribosomal fusion protein** | Solyc08g083120 | 2, 6, 12 14, 15, 20, 29 |
| **LHP1-like** | Solyc10g024470 | 22 |
| **BOBBER 1** | Solyc03g083390 | 26 |
| **CTO99** | Solyc12g013900 | 27 |
| **Coiled-coil domain-containing protein 25** | Solyc08g068300 | 32 |
