# Supplementary material for: Tomato SlSAP3, a member of the stress‐associated protein family, is a positive regulator of immunity against Pseudomonas syringae pv. tomato DC3000
Source: Mol Plant Pathol. 2019 Mar 25;20(6):815–30. doi: 10.1111/mpp.12793 (PMC6637894; doi:10.1111/mpp.12793)
Supplement: Supplementary file 8 — Table S3 Primers used in this study for different purposes. [file MPP-20-815-s008.docx]

**Supplementary Table S3: Primers used in this study for different purposes**

| Primers | Sequences (5’-3’) | Size (bp) |
| --- | --- | --- |
| ***Cloning of cDNA*** | | |
| SlSAP1-F | ATGGCTCAGAGAACGGAGAA | 587 |
| SlSAP1-R | TCAAACTTTAATGATTTTTGCT |  |
| SlSAP2-F | ATGGAGCATGATGAGACAGG | 513 |
| SlSAP2-R | TTAGATCTTGTCAAGTTTTTC |  |
| SlSAP3-F | ATGGAGCATAATGAGACGGGA | 516 |
| SlSAP3-R | CTAGATCTTGTCAAGCTTCT |  |
| SlSAP4-F | ATGGAACAAAATGATACAGG | 504 |
| SlSAP4-R | TCATATTTTGTCAAGCTTTTC |  |
| SlSAP5-F | ATGGAGTCTTCCAAAGAAACA | 519 |
| SlSAP5-R | CTAGATCTTGTTTAGCTTTTCT |  |
| SlSAP6-F | ATGGGTTCTGAAGGCAACAAGT | 498 |
| SlSAP6-R | TTAAAACCGCTGGATTTTATC |  |
| SlSAP7-F | ATGGCGGAAGAACAAAGGATGC | 600 |
| SlSAP7-R | TCAAATTTTGTGTAGCTTTTC |  |
| SlSAP8-F | ATGGCGGAAGAACATGAATTTC | 456 |
| SlSAP8-R | TTAAATCTTCTTTAATTTCTC |  |
| SlSAP9-F | ATGGCGGAAGAACATGGATTTG | 480 |
| SlSAP9-R | TTATATCTTCCCCAATTTCTC |  |
| SlSAP10-F | ATGGCGGCGCAGAAGAGAGAG | 483 |
| SlSAP10-R | TTAAACTTTGAGAATTTTTGCA |  |
| SlSAP11-F | ATGGAAGGAGGAACAGAAGC | 567 |
| SlSAP11-R | TCAACAAGCTTTCACAGAAG |  |
| SlSAP12-F | ATGGGAACGCCAGAGTTCCCA | 819 |
| SlSAP12-R | TTATGCCTTTGAAGTTCCTT |  |
| SlSAP13-F | ATGGGTACACCAGAATTCCCA | 883 |
| SlSAP13-R | TTACATAGAACTTCCTTTATGT |  |
| ***VIGS constructs*** | | |
| SlSAP1-VIGS-F | CCC TCTAGA AGCAGGCAACCGAATTTTGTC | 343 |
| SlSAP1-VIGS-R | GGG CTCGAG CATCCTGTCCAGATCAGACT |  |
| SlSAP2-VIGS-F | CCC TCTAGA AGCAACAATATGGAGCATGAT | 354 |
| SlSAP2-VIGS-R | GGG CTCGAG CTTGCAAGTGCTGCATCGGCT |  |
| SlSAP3-VIGS-F | CCC TCTAGA TTCAAATGTCGCTGTGGTAACC | 326 |
| SlSAP3-VIGS-R | GGG CTCGA CAAAATGAAAAACCTGACAATCG |  |
| SlSAP4-VIGS-F | CCC TCTAGA GCTCAGCTCATCGCTACTCAG | 316 |
| SlSAP4-VIGS-R | GGG CTCGAG ACAAAACAAGCACTAAAGATGG |  |
| SlSAP5-VIGS-F | CCC TCTAGA TTATGGTGTGGCAAGTGAC | 351 |
| SlSAP5-VIGS-R | GGG CTCGAG TCACCTCTGAACTTGGACCT |  |
| SlSAP6-VIGS-F | CCC GAATTC CAAATCAGAAGAAAATCATGG | 337 |
| SlSAP6-VIGS-R | GGG CTCGAG CAAACACCGATTCGGCTGACT |  |
| SlSAP7-VIGS-F | CCC TCTAGA TTCGTTGAGTTAAGAATCACAG | 360 |
| SlSAP7-VIGS-R | GGG CTCGAG CTCAGCTGCAGTATCTGACT |  |
| SlSAP8-VIGS-F | CCC TCTAGA TCCTTTCTCCCTATTTTCAAGA | 372 |
| SlSAP8-VIGS-R | GGG CTCGAG CGTCGTCAAAACAATCGGCT |  |
| SlSAP9-VIGS-F | CCC TCTAGA TGATATCGAAGAGAAATCGA | 364 |
| SlSAP9-VIGS-R | GGG CTCGAG TGAACCCTGTCAATCCCACCT |  |
| SlSAP10-VIGS-F | CCC TCTAGA GCCAAACACTTCCCAACAC | 378 |
| SlSAP10-VIGS-R | GGG CTCGAGA CCCATACCAGAACATCGGT |  |
| SlSAP11-VIGS-F | CCC TCTAGA CACGAAAGAAGAAAATGGAAG | 352 |
| SlSAP11-VIGS-R | GGG CTCGAG GTCAAAATCCCCTTGCATCT |  |
| SlSAP12-VIGS-F | CCC GAATTC TTTGCTTGAAGTTGTTGAGTG | 359 |
| SlSAP12-VIGS-R | GGG CTCGAG AAGAACTCTCTGCAGCCAGGT |  |
| SlSAP13-VIGS-F | CCC TCTAGA GTAGAAAAGTCCTCATTTGCC | 328 |
| SlSAP13-VIGS-R | GGG CTCGAG CGTTGGACACTGATGTCTAATA |  |
| SlBOB1-V-F | CCGGAATTC GCCGTGCTTGATCCTGCTGA | 305 |
| SlBOB1-V-R | CGCGGATCC CCATTCTTGTCCACCTCCTTA |  |
| SlBOBa-V-F | CCG GAATTC ATGGTGATCATTACCGAATACAA | 278 |
| SlBOBa-V-R | CGC GGATCC ATTGCCTCAGTAGCATTATTG |  |
| ***Generation of transgenic tomato /Co-Immunoprecipitation*** | | |
| SlSAP3-OE-HA-F | TT GGCGCGCC ATGGAGCATAATGAGACGG | 516 |
| SlSAP3-OE-HA-R | CGG GGTACC GATCTTGTCAAGCTTCTCTG |  |
| SlBOB1-HA-F | TT GGCGCGCC ATGGCGATTATTTCTGATTTC | 909 |
| SlBOB1-HA-R | CGG GGTACC GGATATCTTTGCCTTTGAGA |  |
| SlBOB1-GFP-F | CGCGGATCC ATGGCGATTATTTCTGATTTC | 909 |
| SlBOB1-GFP-R | TGCTCTAGA TCAGGATATCTTTGCCTTTGA |  |
| SlBOB2-GFP-F | TGC TCTAGA ATGGCAATTCTCTCAGATTATCA | 681 |
| SlBOB2-GFP-R | TCC CCCGGG GCCCATCATCCTAGAATTTGGCA |  |
| SlBOB3-GFP-F | CGCGGATCC ATGGTGATCATTACCGAATAC | 762 |
| SlBOB3-GFP-R | TGCTCTAGA TCGCTGATCAAACATAACAGT |  |
| ***E3 ligase activity*** | | |
| SlSAP3-GST-F | CGC GGATCC ATGGAGCATAATGAGACGGGA | 516 |
| SlSAP3-GST-R | GCG CTCGAG CTAGATCTTGTCAAGCTTCTCT |  |
| ***Yeast transformation*** | | |
| SlSAP3-A20-BD-F | CCG GAATTC ATGGAGCATAATGAGACGG | 171 |
| SlSAP3-A20-BD-R1 | CGCGGATCC GTTTTCAATTGATGATACAGC |  |
| SlSAP3-AN1-BD-F1 | CCGGAATTC GCACAGTCTATAGCTTTGC | 267 |
| SlSAP3-AN1-BD-R | CGC GGATCC CTAGATCTTGTCAAGCTTCTCTG |  |
| SlSAP3-ΔA20AN1-BD-F2 | CCGGAATTC GGATCGTCTGCCAGTGAG | 135 |
| SlSAP3-ΔA20AN1-BD -R2 | CGCGGATCC ACCCACCTTAGCCTTTACA |  |
| SlBOB1-AD-F | CCGGAATTC ATGGCGATTATTTCTGATTTC | 909 |
| SlBOB1-AD-R | CGCGGATCC GGATATCTTTGCCTTTGAGA |  |
| SlBOB2-AD-F | CCG GAATTC ATGGCAATTCTCTCAGATTATC | 681 |
| SlBOB2-AD-R | CGC GGATCC CTAGCCCATCATCCTAGAAT |  |
| SlBOB3-AD-F | CCG GAATTC ATGGTGATCATTACCGAATAC | 762 |
| SlBOB3-AD-R | CGC GGATCC TCGCTGATCAAACATAACAGT |  |
| ***Bimolecular Fluorescence Complementation*** | | |
| SlSAP3-P2YC-F | CCCTTAATTAAC ATGGAGCATAATGAGACGG | 516 |
| SlSAP3-P2YC-R | GGGACTAGT GATCTTGTCAAGCTTCTCTG |  |
| SlBOB1-P2YN-F | CCCTTAATTAAC ATGGCGATTATTTCTGATTTC | 909 |
| SlBOB1-P2YN-R | GGGACTAGT GGATATCTTTGCCTTTGAGA |  |
| SlBOB2-P2YN-F | CCC TTAATTAAC ATGGCAATTCTCTCAGATTATCA | 681 |
| SlBOB2-P2YN-R | GGG ACTAGT GCCCATCATCCTAGAATTTGGCA |  |
| SlBOB3-P2YN-F | CCCTTAATTAAC ATGGTGATCATTACCGAATACAA | 762 |
| SlBOB3-P2YN-R | GGGACTAGT TCGCTGATCAAACATAACAGT |  |
| ***qRT-PCR*** | | |
| SlSAP1-RT-F | GGAAGGTAGGATTGACCGGATT | 68 |
| SlSAP1-RT-R | AGTATCGATGTTCACCGCAAAA |  |
| SlSAP2-RT-F | ACCGCTACTCAGATAAACATGACTGT | 71 |
| SlSAP2-RT-R | GGCCTTTGCAATAGCATTGC |  |
| SlSAP3-RT-F | TGCCTGTTTGATTACCGCTCTG | 82 |
| SlSAP3-RT-R | TCTTGTCAAGCTTCTCTGCCTT |  |
| SlSAP4-RT-F | GCAAGGCTGGTCAAGATGCT | 75 |
| SlSAP4-RT-R | TCCTTCATATTTTGTCAAGCTTTTCA |  |
| SlSAP5-RT-F | AGGGCCCAACTAGGTGCACT | 77 |
| SlSAP5-RT-R | GAAAAGATTCCCACACTTGCAA |  |
| SlSAP6-RT-F | GCTGCTAAACCAAAATTAAGTTG | 101 |
| SlSAP6-RT-R | CTACTTTTCATACACATCCAATCCTC |  |
| SlSAP7-RT-F | GTGCCACGTGTCGAAAACG | 94 |
| SlSAP7-RT-R | CCATGATGCTCAGGGTACCTATG |  |
| SlSAP8-RT-F | CCTAACCGTTGGATTAGATGACTTTTT | 77 |
| SlSAP8-RT-R | CACCATCATCATCGCTTTCGT |  |
| SlSAP9-RT-F | ATCGGAAGAGAAGCCATTGCT | 73 |
| SlSAP9-RT-R | GCTGTGATTATATCTTCCCCAATTTC |  |
| SlSAP10-RT-F | AGGGTGGGTGGGAAATGAA | 73 |
| SlSAP10-RT-R | ATGACAACAATACCCTCATCCATTC |  |
| SlSAP11-RT-F | GTTTTTGACTGCTTTGCTTGCA | 76 |
| SlSAP11-RT-R | CGCAGGGCTTGGAGATGA |  |
| SlSAP12-RT-F | CAGCATTCTCCTTTATGAACTTTCG | 73 |
| SlSAP12-RT-R | GATGACGTGGCTGGAGCTTTA |  |
| SlSAP13-RT-F | GGTAGTATCATCTGTAAAGGAAAAG | 75 |
| SlSAP13-RT-R | TGGTGGCGCGGCTGCTTTGC |  |
| SlBOB1-RT-F | AAAGTCCATCTCTGTTCTCCTA | 129 |
| SlBOB1-RT-R | GGGTCCAAATCTGAAAGCTTAC |  |
| SlBOB2-RT-F | GGACACAGAAACAAGAGCAGC | 82 |
| SlBOB2-RT-R | CTCCTCACTTGATGGAAGTCC |  |
| SlBOB3-RT-F | CCTCAGGGAACAAAATCACGG | 91 |
| SlBOB3-RT-R | CATCAACTATTGGAGGCTGACC |  |
| SlActin-RT-F | CCAGGTATTGCTGATAGAATGAG | 113 |
| SlActin-RT-R | GAGCCTCCAATCCAGACAC |  |
| SlPI-II-RT-F | AATTTATCCCACCGGATGTACC | 158 |
| SlPI-II-RT-R | GGTTCATCACTCTCTCCTTCAC |  |
| SlPR1a-RT-F | GGCAGGAACACCAAAGAAACCA | 127 |
| SlPR1a-RT-R | TGGCCTCTGGTCAGGTTTAAAG |  |
| SlPR1b-RT-1F | CCGTGCAATTGTGGGTGTC | 106 |
| SlPR1b-RT-1R | GAGTTGCGCCAGACTACTTG |  |
| SlPR-P2-RT-F | CGATCTAAATTGATTTCATAGTACG | 116 |
| SlPR-P2-RT-R | TCGTGAAGGATATACAAAATACA |  |
| SlEDS1-RT-F | GAATGACCTTGGCCTGAGTACAAG | 114 |
| SlEDS1-RT-R | CCTGCTGCACGAAGACACAG |  |
| SlTGA1-RT-F | GGCATGTGGGATGATTTC | 116 |
| SlTGA1-RT-R | CATCATCGGTATCTGGTCCT |  |
| SlJAZ1-RT-F | GATTTTCCGGCTGATAAAGCTAA | 114 |
| SlJAZ1-RT-R | TCCGAAACTCGGAACCACCAA |  |
| SlACS1-RT-F | GTGCTTCAAACAAAGGGAC | 146 |
| SlACS1-RT-R | GTCCTAACCAAAGGCGAAT |  |
| SlERF1-RT-F | TGGAGTTAGAAAGAGGCCATGG | 143 |
| SlERF1-RT-R | CCCTCATTGATAATGCGGCTTG |  |
| SlPTI5-RT-F | ATTCGCGATTCGGCTAGACATGGT | 95 |
| SlPTI5-RT-R | AGTAGTGCCTTAGCACCTCGCATT |  |
| SlLRR22-RT-F | AAGATTGGAGGTTGCCATTGGAGC | 100 |
| SlLRR22-RT-R | ATCGCGATGAATGATCGGTGGAGT |  |
| SlWRKY28-RT-F | ACAGATGCAGCTACCTCATCCTCA | 125 |
| SlWRKY28-RT-R | GTGCTCAAAGCCTCATGGTTCTTG |  |
