# Supplementary material for: Tomato SlSAP3, a member of the stress‐associated protein family, is a positive regulator of immunity against Pseudomonas syringae pv. tomato DC3000
Source: Mol Plant Pathol. 2019 Mar 25;20(6):815–30. doi: 10.1111/mpp.12793 (PMC6637894; doi:10.1111/mpp.12793)
Supplement: Supplementary file 9 — Table S4 CDS and amino acid sequences of the SlSAP and SlBOB genes. [file MPP-20-815-s009.docx]

**Supplementary Table S4: CDS and amino acid sequences of the *SlSAP* and *SlBOB* genes**

**>*SlSAP1* CDS (Soly****c01g086960)**

ATGGCTCAGAGAACGGAGAAAGAAGAGACGGAGTTCAAGGCCGTACCTGAAACGATAACGCTTTGCATCAACAATTGTGGAGTTACAGGAAATCCAGCCACAAACAATATGTGTCAAAAGTGCTTCAACGCCACCACCGCGGCTACCTCAACTTCATCCAGCTCGCCGACGGGGACGTCGGTGACGATTCCTCACAATTTCGCTGAAAAATTGGTCAGATCTGAAAAATCGGCGAGATTCAGCTCGTTGAGGTCGTCGCCGGACAGGAAGTCTGATCTGGACAGGATGAGTCAAGATCTGAAGAAAGTTGGAGATACGATGATGGTAAAGGAGGAGGATCAACTGAAGGCAAGCTTACCGCCGGCTAAGAGAGAGGTGAATCGTTGCTCCGGTTGTCGAAGGAAGGTAGGATTGACCGGATTCCGATGCCGGTGCGGCGAGTTGTTTTGCGGTGAACATCGATACTCCGATCGTCATGATTGTAGCTACGACTACAAAACCGCCGGCCGAGAAGCGATCGCGAGGGAGAATCCAGTCGTGAAAGCAGCAAAAATCATTAAAGTTTGA

**> SlSAP1 Protein**

MAQRTEKEETEFKAVPETITLCINNCGVTGNPATNNMCQKCFNATTAATSTSSSSPTGTSVTIPHNFAEKLVRSEKSARFSSLRSSPDRKSDLDRMSQDLKKVGDTMMVKEEDQLKASLPPAKREVNRCSGCRRKVGLTGFRCRCGELFCGEHRYSDRHDCSYDYKTAGREAIARENPVVKAAKIIKV

**>*SlSAP2* CDS (Solyc11g061770)**

ATGGAGCATGATGAGACAGGATGTCAACCCCATCCAGAAGGCCCTATTTTGTGCATTAACAATTGTGGGTTTTTTGGAAGTGCTGCGAACATGAATATGTGCTCCAAGTGTTATAAGGACGTGATTCTGAAACAAGAACAAGAAAAGCTTGCAGCATCGTCAATTGAAAACTTTGTGAATGGGTCAACAAGTCAGAAGGGGCCTGTCATTGTTGGCTCTGTGGATGTGCAACCTGCCCTGCTAGAATCAAAATCTGTGGTTTTGTCATCACCTCCATCTTCAAGTTCTGGTGAGGCTGCTGAATTGATGGCCAAGGAGGGTCCTAGCCGATGCAGCACTTGCAAGAAAAAGGTTGGTTTGACTGGATTCAAATGCCGCTGTGGTAACTTTTACTGTGGATCACACCGCTACTCAGATAAACATGACTGTCAATTTGACTACCGCTCTGCTGCACGCAATGCTATTGCAAAGGCCAATCCTGTTGTGAAGGCAGAAAAACTTGACAAGATCTAA

**>SlSAP2 Protein**

MEHDETGCQPHPEGPILCINNCGFFGSAANMNMCSKCYKDVILKQEQEKLAASSIENFVNGSTSQKGPVIVGSVDVQPALLESKSVVLSSPPSSSSGEAAELMAKEGPSRCSTCKKKVGLTGFKCRCGNFYCGSHRYSDKHDCQFDYRSAARNAIAKANPVVKAEKLDKI

**>*SlSAP3* CDS (Solyc01g014180)**

ATGGAGCATAATGAGACGGGATGCCAACCCCCTCGTGAAGGTCCTATTTTGTGTATTAACAACTGTGGCTTTTTTGGAAGTGCTGCAAACATGAACATGTGCTCCAAGTGTTACAAAGACATGGTATTGAAACAAGAACAAGCTAAGCTTGCTGTATCATCAATTGAAAACCTTGTCAATGGATCGTCTGCCAGTGAGAAGGGAATGGTTATTGCTGGCCCCGTAGATGTGCAACCTGATACTATTGAAGCACAGTCTATAGCTTTGCCATCATCTCAAACTTCAAGCTCTAGTGATATGCCCGATGTAAAGGCTAAGGTGGGTCCTAACCGCTGTGGCACTTGTAAGAAAAAGGTTGGCATCACTGGATTCAAATGTCGCTGTGGTAACCTTTATTGTGGAGCACACCGCTACTCAGATAAACATGACTGCCTGTTTGATTACCGCTCTGCTGGCCAGGATGCAATAGCGAAGGCCAACCCTGTTGTTAAGGCAGAGAAGCTTGACAAGATCTAG

**>SlSAP3 Protein**

MEHNETGCQPPREGPILCINNCGFFGSAANMNMCSKCYKDMVLKQEQAKLAVSSIENLVNGSSASEKGMVIAGPVDVQPDTIEAQSIALPSSQTSSSSDMPDVKAKVGPNRCGTCKKKVGITGFKCRCGNLYCGAHRYSDKHDCLFDYRSAGQDAIAKANPVVKAEKLDKI

**>*SlSAP4* CDS (Solyc01g086970)**

ATGGAACAAAATGATACAGGCTGCCAAGCTCCTCAAGCTCCTGTCCTTTGTGTTAACAATTGTGGGTTTTTTGGTACTGCAGCAACAATGAACATGTGCTCAAAGTGCTACAAGGATATGATATTCAAGCAAGAACAAGCAAACTTTGCTGCTTCATCTATTGAAAGCTTTGTAAATGGAAGTTCAAATGCTAGTGTGAAAGCTGTTGATGTTGCTGTCACCGTGCAGGAAGGTCCTGCAGAGTCCCTGGTTATACCCACACAAGTCGCGGTACCAGTAGAGAGTGAACAAGTTGAGAAGGCTAAGGAGGGGCCAAACAGGTGCAGCACCTGTAGGAGACGAGTTGGTTTGACCGGCTTCAACTGCCGATGTGGGAATCTTTTCTGCTCAGCTCATCGCTACTCAGACAAACATGAGTGTCCATATGATTATCGCAAGGCTGGTCAAGATGCTATTGCGAAAGCCAACCCAGTTGTTAAGGCTGAAAAGCTTGACAAAATATGA

**>SlSAP4 Protein**

GCQAPQAPVLCVNNCGFFGTAATMNMCSKCYKDMIFKQEQANFAASSIESFVNGSSNASVKAVDVAVTVQEGPAESLVIPTQVAVPVESEQVEKAKEGPNRCSTCRRRVGLTGFNCRCGNLFCSAHRYSDKHECPYDYRKAGQDAIAKANPVVKAEKLDKI

**>*SlSAP5* CDS (Solyc10g079080)**

ATGGAGTCTTCCAAAGAAACAGGCTGTCAAGCTCCCGAAGGTCCCATCCTATGCATCAACAACTGCGGGTTCTTTGGTAGTGCAGCCACTATGAATATGTGTTCCAAGTGTCACAAGGACATGATACTGAAGCAGGAACAAGCTAAATTTGCAGCAACATCAATTGAAAACATAGTAAATGGAAACTCAAGCAGCAATGGCAAAGAGCCTATTGCGACTGGTGCAATCAATGTTCAACCGGGATCAGCAGACTTGAAGGTTATCTCTACAGAAGCATCTTCTGATTTATCTTCAGGTCCAAGTTCAGAGGTGAAGCCAAAAGAGGGCCCAACTAGGTGCACTACTTGCCGCAAGCGTGTTGGCTTGACAGGATTCAATTGCAAGTGTGGGAATCTTTTCTGTGCGGCTCACCGTTATTCTGACAAGCACGAGTGCCCATTTGACTATAAGAATGCTGGACGGGATGCTATTGCTAAAGCAAATCCTGTTGTTGTAGCAGAAAAGCTAAACAAGATCTAG

**>SlSAP5 Protein**

MESSKETGCQAPEGPILCINNCGFFGSAATMNMCSKCHKDMILKQEQAKFAATSIENIVNGNSSSNGKEPIATGAINVQPGSADLKVISTEASSDLSSGPSSEVKPKEGPTRCTTCRKRVGLTGFNCKCGNLFCAAHRYSDKHECPFDYKNAGRDAIAKANPVVVAEKLNKI

**>*SlSAP6* CDS (Solyc08g079700)**

ATGGGTTCTGAAGGCAACAAGTTTAACGATGGAACAAGCTTCCCGCCGGCGGATCCGATTCTCTGTTCAAACGGTTGCGGATTTTTTGGTACGGCGGCGACGAATGGACTTTGTTCAAAGTGTTACCGTGACTTTAAGATGAAGGAAGATCATGCTGCGATGGCTAAAGTAGCGATGGAGAAACTTGTTATATCTAGACCTCAAATTGAATCGATCGGAAAAGTTGATTTCTGTTCGTCGACTACATCGACGGCTGCGGAGAGGCCGGTTGTTGAGGCGGCGACGGCGGAGATCGGCGGGAGTCAGCCGAATCGGTGTTTGAGCTGTAGGAAGAAGGTGGGATTTGTGGGATTTAAGTGCAGATGTGGTAGTACCTTTTGTGGGACCCATAGATACCCGGAGAAACATGATTGTACCTTTGATTTTAAAATCAAAGGGAAGGAAGAGATTTGTAAGGCTAATCCTGTAGTTAAGGCTGATAAAATCCAGCGGTTTTAA

**>SlSAP6 Protein**

FPPADPILCSNGCGFFGTAATNGLCSKCYRDFKMKEDHAAMAKVAMEKLVISRPQIESIGKVDFCSSTTSTAAERPVVEAATAEIGGSQPNRCLSCRKKVGFVGFKCRCGSTFCGTHRYPEKHDCTFDFKIKGKEEICKANPVVKADKIQRF

**>*SlSAP7* CDS (Solyc07g055090)**

ATGGCGGAAGAACAAAGGATGCAAGAAGGAGGAGGACATAGGCTATGTGCTAATAATTGTGGTTTCTTTGGTAGCCCAACAACTTTGAACCTTTGTTCCAAATGCTACAAGGATCATTGCATGAAAGAACAACAATCGCGAACCGCTCAGCTCGCAATGGAAAAGACTCGTCCCCAACAACAACAACAACAACAATCTGAATCAACGTCTACGTACATACCATGTACAAAGCCGTTACCAATTCTTGAAGTCTCACAACCACGAGAGACAGAGATTGCAACTAGGGCTCCTCAGGTGCAGTCAGATACTGCAGCTGAGGTTCCTCAGGTGCAATCAGATACTGTAGCTGAGGTTCCTCAGGTGCATACACAATTAAATGATGTAGCTGATCAAGCTCCTCAAGTGCAGTCAAATCGTTGTGCCACGTGTCGAAAACGGGTCGGTTTAACGGGTTTCAAGTGTAGGTGTGGGGTCACATTTTGTGGTTCACATAGGTACCCTGAGCATCATGGTTGCACCTTTGACTATAAGTCAATGGGAAAAATGGCAATTGCCATGGCTAATCCATTGGTTAAGGCTGAAAAGCTACACAAAATTTGA

**>SlSAP7 Protein**

MAEEQRMQEGGGHRLCANNCGFFGSPTTLNLCSKCYKDHCMKEEQSRTAQLAMEKTRHQQQQQQQSESTSAYIPCTEPLSILEVSQPRETEIATRAPHVQSDTAAEVPQVQSDTVAEVPQVHTQLNDVADQAPQVQSNRCATCRRRVGLTGFKCRCGVTFCGSHRYPEHHGCTFDYKSMGKMAIAMANPLVKAEKLHKI

**>*SlSAP8* CDS (Solyc09g009590)**

ATGGCGGAAGAACATGAATTTCAATCCCAAGAAGGTGGACGTCACCAATTATGTGCAAATAATTGTGGTTTTTTTGGCAACTCTACAACCGAAAATTATTGTTCAAAATGTTATCGCGATATCGAAAAACAAAAATCCGATGCAAAATCAATCGATTCTCTTTTTTCTCCGATAAAAAAGGTTTCGGAAAAAAAGATAATCGAGCCGATTGTTTTGACGACGGATACAATGAAAACTACGACGTCAAATGTTGTGACGCCGCAGTCCAATAGATGTTTGGTTTGTAAGAAGAAAATGGGGTTAATGGGATTTAGATGCAAGTGTGGGACCATATTTTGTGGGACCCATAGGTATCCAGAGGTTCATGCTTGTACATTTGATTTTAAGTCAATGGGGAGAGAAGCTATAGCTAAGGCAAATCCATTGATCAAAGCTGAGAAATTAAAGAAGATTTAA

**>SlSAP8 Protein**

MAEEHEFQSQEGGRHQLCANNCGFFGNSTTENYCSKCYRDIEKQKSDAKSIDSLFSPIKKVSEKKIIEPIVLTTDTMKTTTSNVVTPQSNRCLVCKKKMGLMGFRCKCGTIFCGTHRYPEVHACTFDFKSMGREAIAKANPLIKAEKLKKI

**>*SlSAP9* CDS (Solyc10g083460)**

ATGGCGGAAGAACATGGATTTGAAGCACCAGAAGGACATATATTATGCGCTAATAACTGTGGTTTCTTTGGTAGCCCAACAACGCAAAATTTCTGCTCCAAATGTTACAATGAAGTTTACATAAAAGGGGGACAACAAAAACCTATTGATTCCCTTTTTCCTCCTTCGCAGCTGCCGATTCCATCAACCTCATCGATACTGGTGCTGCAGGAATCGACAGCTGCGGAGGAAGAACCTGAGGTTGTGACCGCGGCTGTAACAGTCGCGGTCCAACCGATTTCTGCACAGCCGAACAGATGTTCGGCCTGCAGGAAGAAGGTGGGATTGACAGGGTTCAAGTGCAGATGTGGGACCACGTTTTGTGGGACCCATCGGTACCCGGAGATCCATGGATGCTCGTTTGATTTCAAATCTATCGGAAGAGAAGCCATTGCTAAGGCGAATCCGGTGGTTAAAGCAGAGAAATTGGGGAAGATATAA

**>SlSAP9 Protein**

MAEEHGFEAPEGHILCANNCGFFGSPTTQNFCSKCYNEVYIKGGQQKPIDSLFPPSQLPIPSTSSILVLQESTAAEEEPEVVTAAVTVAVQPISAQPNRCSACRKKVGLTGFKCRCGTTFCGTHRYPEIHGCSFDFKSIGREAIAKANP

**>*SlSAP10* CDS (Solyc12g100060)**

ATGGCGGCGCAGAAGAGAGAGAAAGAAGAAACCGAGCTGAAGGTGCCGGAATCCATCCCTCTATGCTCTCCAACTCTACCGGTACCTTCACCATCGCCTCCTTCTACGACGACGCACCTCTCAGTTGCTGTTATCTCAAATCTGAAGCGTTCTGATAGATCGTCGACGGAGAGTATAGATCTGAAGGTTTCTAGTATGGATGATCAATCGAGATCTACATCAGCTGCATCGCCGGAAAGTATGGATCTGGTTGGTAGAAAAACAGGGGTTAAGAGGCAAAGAGAAGCGAACCGATCTTCTGGTATGGGTTGCCGGAGGAAAGTCGGATTGATGCCGTTCCGGTGCCGGTGTGGGGAAGTGTTCTGCTCGGAGCATAGGTACTCCGATCGACATGACTGTAGTTACGATTACAAAGCAGCTGGTCGTGAAGCGATTGCTAAGGAAAATCCAGTGGTGAAAGCTGCAAAAATTCTCAAAGTTTAA

**>SlSAP10 Protein**

MAAQKREKEETELKVPESIPLCSPTLPVPSPSPPSTTTHLSVAVISNLKRSDRSSTESIDLKVSSMDDQSRSTSAASPESMDLVGRKTGVKRQREANRSSGMGCRRKVGLMPFRCRCGEVFCSEHRYSDRHDCSYDYKAAGREAIAKENPVVKAAKILKV

**>*SlSAP11* CDS (Solyc02g087210)**

ATGGAAGGAGGAACAGAAGCCTATCCAGATTTAGGTAGACATTGCCAAATATCTGATTGCCATCAAATCGATTTTCTCCCTTTTACCTGCCATGCCTGTTTAAAGGTATTTTGTGTGGAACATAGATCATGCAAGTCTCATGAATGCCCAAAATCTGACTTTAACAGCCGAATCGTTTTGGTTTGCGAAATTTGTTCTATGTCCATGGAAACTACCGGCTGTAAAGTTGAAGACCACAAAGCAATATTACAAAAACACGAGGAATCTGGGGATTGTGACCCTAAGAAGAAGAAGAAGAAACCTACCTGTCCTGTCAAAAGATGCAAGGGGATTTTGACCTTCTCAAACACTAACACTTGCAAGATATGCCGGATTCAAGTTTGCCTCAGACACAAGTTCCCTGCTGATCACGCCTGTGACCCCACTTCTTCATCATCACAGCTGTTGCTAAAGGAACCCAATAACAAGTTTTTGACTGCTTTGCTTGCAAGGAATGGGAAAGATTGTGGGAATAAAAGTCGTGCCTCATCTCCAAGCCCTGCGAACCCTTCTGTGAAAGCTTGTTGA

**>SlSAP11 Protein**

MEGGTEAYPDLGRHCQISDCHQIDFLPFTCHACLKVFCVEHRSCKSHECPKSDFNSRIVLVCEICSMSMETTGCKVEDHKAILQKHEESGDCDPKKKKKKPTCPVKRCKGILTFSNTNTCKICRIQVCLRHKFPADHACDPTSSSSQLLLKEPNNKFLTALLARNGKDCGNKSRASSPSPANPSVKAC

**>*SlSAP12* CDS (Solyc10g080200)**

ATGGGAACGCCAGAGTTCCCAAATCTTGGAAAACATTGCTTTGTAGATGACTGCAGGCAGATTGATTTCTTGCCTTTTACCTGCGATTGTTGTCACCAGGTCTTTTGTCTAGAGCATCGGAGCTATAATAGACACCACTGTCCGACAGCGAACAATAATGATGTTACTGTGGTTGTTTGCCCACTCTGTGCAAAAGGAGTACACCTTATTCCTGACGAAGACCCAAATATAACTTGGGAATCACATGTAAACACAGATTGTGATCCATCAAACTACGAAAAAGCCACAAAGAAAAGAAAATGTCCCGTACCTGGCTGCAGAGAGTTCTTGACTTTCTCAAACACAATCAGATGTCGGGAGTGTACTGTAGATCATTGTTTGAAGCATCGTTTTGGACCTGATCACAAATGTCCTGGACGTAAGAAACCAGAAGCAGCATTCTCCTTTATGAACTTTCGCACTGGCAGTAGAAATGGCGAGCCTAATAAAGCTCCAGCCACGTCATCCTCTAGTTGGGCCTCATCATTCTTCAAGGCAGCTGAAGCTGGAATGGCAAAATTAGGCAGCGGAAGGGGCCAAAGCAGCAATGCCACAAACCATAGTGGGAGTGCTAACAGGCAAGTTGAGCAATGCCCGCAATGCACTCTTAGATTCTCTTCAGTCACAGCTCTCGTGAGCCACGTACAAAAAGTCCATGAAAAAAACGATGTCATGAACTTGACAGTCGATGTCTGCCCAAGATGTAGTAAAGGCTTTCGCGATCCAGTGTCCCTTGTAGAACATGTTGAAAGGGAACATAAAGGAACTTCAAAGGCATAA

**>SlSAP12 Protein**

PLCAKGVHLIPDEDPNITWESHVNTDCDPSNYEKATKKRKCPVPGCREFLTFSNTIRCRECTVDHCLKHRFGPDHKCPGRKKPEAAFSFMNFRTGSRNGEPNKAPATSSSSWASSFFKAAEAGMAKLGSGRGQSSNATNHSGSANRQVEQCPQCTLRFSSVTALVSHVQKVHEKNDVMNLTVDVCPRCSKGFRDPVSLVEHVER

**>*SlSAP13* CDS (Solyc04g015570)**

ATGGGTACACCAGAATTCCCAAATCTTGGGAAGCATTGTTCTGTTGAGGATTGTAGGCAGATTGATTTCTTGCCTTTTACCTGTGATTGCTGTTTCAAGGTGTATTGTTTAGATCATCGAAGCTATATTAGACATCAGTGTCCAACGGCTAACAAGAATAATGTTACAGTGGTCATTTGCCCCCTCTGTGCAAAAGGGGTACGCCTAAATCCTGTCGAAGACCCAAATATAACTTGGGAATCGCATGTGAACACCGAGTGTGATCCATCGAACTATGAGAAAGCCACAAAGAAAAAAAAATGTCCTGTGCCTCGCTGCAAAGAGCTTTTGACTTTCTCCAACACAATCAAATGTCGGGATTGTACTATCGATCATTGCTTGAAGCACCGGTTTGGACCTGATCACAGCTGTGCAGGACCTAAGAAACCAGAAGCTACTTTCCAGTTCATGAACTTTCTGAATGGAAGTAAAGAGGATTCGAAGAAAGCTCAGCCCACGACGACCTCAAGGTGGACCACGAGCCTTTTAAAGGTAGTATCATCTGTAAAGGAAAAGTTCAACAACGAATTTAATCAACCACAGCAGATGGGGCAAAGCAGCCGCGCCACCAACCATAGTGTTACTAACAACAGCAGTCAAGTAGAGCCATGCCCACAATGTCATCTGAGATTTTCTACAGTCAGAGCTCTCATCGACCACGTGCAGAAAGTACACGAAAAGAATGGTGTTATGAACATGACAATCGATGTCTGCCCGAGGTGTAGTAAAGGTTTTCGAGATCCTGTTGCCCTTGTGGAACATGTTGAAAGGGAACATAAAGGAAGTTCTATGTAA

**>SlSAP13 Protein**

MGTPEFPNLGKHCSVEDCRQIDFLPFTCDCCFKVYCLDHRSYIRHQCPTANKNNVTVVICPLCAKGVRLNPVEDPNITWESHVNTECDPSNYEKATKKKKCPVPRCKELLTFSNTIKCRDCTIDHCLKHRFGPDHSCAGPKKPEATFQFMNFLNGSKEDSKKAQPTTTSRWTTSLLKVVSSVKEKFNNEFNQPQQMGQSSRATNHSVTNNSSQVEPCPQCHLRFSTVRALIDHVQKVHEKNGVMNMTIDVCPRCSKGFRDPVALVEHVEREHKGSSM

**>*SlBOB1* CDS (Solyc03g083390)**

ATGGCGATTATTTCTGATTTCGAGGAGCAAGATAGCAAACCATCGGCGGCGGCGGCGGCGTCAAAGCCCTTTAAGGCCGTGCTTGATCCTGCTGATCCTCTAGGGTTTCTTCAAGCCGCGTTCGAGTTCGTCGGACGGGAATCTGATCTTTTCAAGAGTGATTCATTGATTAATGATGTCAATGCTGTTGTTCGTATGGTGAAAGACAAGCTGTTGACTGAGGAGCGCAAGAGGAAAGTGGAAGCAGAAGCATCAAGCTCAAAGGCGGCGGGAAAGAAGGTCAAGGAGGATGTTCCAGTTGCTGCTGCAAAGAAGGAAGAGGTTAAAGAGGCAAAAGGGAAAGAAGTTATGAAGGAGGCTAAGGAGGTGGACAAGAATGGTACTCAAGGTCCTGCAGCTCCTAACAACAACAATGGCCTTGACCTTGAGAACTACTCATGGGGTCAATCACTACAGGAAGTAAATGTTAATATTCCTGTGCCTCCTGGAACCAAATCAAGGTTAATTGTTTGTGACATATCAAGGAACCGTATTAAAGTTGGATTAAAGGGACAGCCTCCAATAATTGATGGAGAACTATATCGACCTGTGAAAGTTGATGATTGTTTCTGGAGTTTAGAGGACCAAAAGTCCATCTCTGTTCTCCTAACCAAGAAGGACCAAATGGAATGGTGGAAATGCTGTGTGAAAGGTGAACTGAAATTGATACGCAGAAAGCAGAACCTGAAAGTAGTAAGCTTTCAGATTTGGACCCCGAGACGCGATCAACTGTGGAAAAGATGATGTTTGATCAGCGACAGAAATCCATGGGTCTTCCAACAAGTGATGAGACGCAGAAACAAGAAATTCTCAAGAAATTTATGGCAGAGCATCCTGAAATGGACTTCTCAAAGGCAAAGATATCCTGA

**>SlBOB1 Protein**

MAIISDFEEQDSKPSAAAAASKPFKAVLDPADPLGFLQAAFEFVGRESDLFKSDSLINDVNAVVRMVKDKLLTEERKRKVEAEASSSKAAGKKVKEDVPVAAAKKEEVKEAKGKEVMKEAKEVDKNGTQGPAAPNNNNGLDLENYSWGQSLQEVNVNIPVPPGTKSRLIVCDISRNRIKVGLKGQPPIIDGELYRPVKVDDCFWSLEDQKSISVLLTKKDQMEWWKCCVKGEPEIDTQKAEPESSKLSDLDPETRSTVEKMMFDQRQKSMGLPTSDETQKQEILKKFMAEHPEMDFSKAKIS

**>*SlBOB2* CDS (Solyc02g062410)**

ATGGCAATTCTCTCAGATTATCAAGAAGAAAATCAAGAACAAACTATGGAGATTATTGAGAAAGAACAAGATAATTCTTCTACTTTAGCCCCTAAAGAAGAAGAAATTTCATCTTCAACCCCAAAAGAAGAGAAACTTAAGCCAAATAAGTCCAATGGACTTGATATGGAAAATTATTCATGGGGACAATCTCTTCAAGAAGTTACCATCAATGTTCCTGTTCCTCCAGGTACAAAATCAAGATTCATAATTGTGGAAATCAAGGCCAATACTCTCAAAGTTGGTCTAAAAAACCAACCACTAATATTAGATGGTGAATATTTCAAGGGAGTTAAAGTTGATGAATGTTATTGGAGTTTAGAAGATGAAAAGGAAATTTCAATTCTTTTAACAAAACAAAACAAAACTGATTGGTGGAAAAGTTTATTTAAAGGTGGAGAAGAAATTGACACACAAAAAGTAGAGCCAGAACCAAGTAAATTGAGTGATTTGGACACAGAAACAAGAGCAGCAGTTGAAAAAATGATGTTTGATCAAAGACAAAAACAAATGGGACTTCCATCAAGTGAGGAGATTAAGAATCAAGATATGCTTAAACAATTTATGGAACAAAATCCTCATATGGCTAAGAATTTTGGGAATGCTAACATGATGATGCCAAATTCTAGGATGATGGGCTAG

**>SlBOB2 Protein**

MAILSDYQEENQEQTMEIIEKEQDNSSTLAPKEEEISSSTPKEEKLKPNKSNGLDMENYSWGQSLQEVTINVPVPPGTKSRFIIVEIKANTLKVGLKNQPLILDGEYFKGVKVDECYWSLEDEKEISILLTKQNKTDWWKSLFKGGEEIDTQKVEPEPSKLSDLDTETRAAVEKMMFDQRQKQMGLPSSEEIKNQDMLKQFMEQNPHMAKNFGNANMMMPNSRMMG

**>*SlBOB3*** **CDS (Solyc06g051950)**

ATGGTGATCATTACCGAATACAATGAGCAAGATGACATACCACCATCGTCCTCACCTGTAGAGGAGGTTAAGGACAAGAACGCTACTAAGGCCATTGAAGAGAACAAGAAGGGGCTTCAAGCTCCCAACATAGGCAATGTCCTTGATCTGGACAACTACTCTTGCGGTCAATCCGTACAGGAGGTTAAGGTGGCCAATAATGCTACTGAGGCAATTGAAGAGAACAAGAAGGGGCATCTAGCTCCCACCATAGGCAATGGCCTTGATCTGGACAACTACTCTTGCGGTCAATCTGTACAGGAGGTTAAGGAGGCCAATAATGCTACTGAGGTCCTTGAAGAGAACAAGAAGGGGCCTCTAGCTCCCAACATAGACAATGGCCTTGATCTGGACAACTACTGTTGGGGTCAATCATTACAGGAGGTTTATGTCAATATTCTTGTACCTCAGGGAACAAAATCACGGTTTATTGTTTGCGATATAAAAAGGAACCATCTTACAGTTGGACTAAAGGGTCAGCCTCCAATAGTTGATGGAGAACTCTATGGACCTGTCAAAGTTGAGGCTTGTTTCTGGACCTTAGAGGATCAGAAATCCATCTCTCTACTCCTTACCAAGATAGACCAGATGAACTGGTGGAAATGGGTAGTTAAAGGTGAACCTGAACTCGATACCGAGAAAGTTGAACAAATTACAAGCCTATCAGAATTGGACCCGGAGACTCGATCTAGTGTTGAAACTGTTATGTTTGATCAGCGATAG

**>SlBOB3 Protein**

MVIITEYNEQDDIPPSSSPVEEVKDKNATKAIEENKKGLQAPNIGNVLDLDNYSCGQSVQEVKVANNATEAIEENKKGHLAPTIGNGLDLDNYSCGQSVQEVKEANNATEVLEENKKGPLAPNIDNGLDLDNYCWGQSLQEVYVNILVPQGTKSRFIVCDIKRNHLTVGLKGQPPIVDGELYGPVKVEACFWTLEDQKSISLLLTKIDQMNWWKWVVKGEPELDTEKVEQITSLSELDPETRSSVETVMFDQR
